# Characterization of synaptopodin in striated and smooth muscles: isoform spectrum, expression patterns, localization and protein interactions

**DOI:** 10.1016/j.yexcr.2026.114990

**Published:** 2026-04-04

**Authors:** Keerthika Lohanadan, Marvin Assent, Anja Linnemann, Chengqing Qu, Anne Schänzer, Lukas Heukamp, Hendrik Milting, Gregor Kirfel, Jeffrey H. Miner, Dieter O. Fürst, Peter F.M. van der Ven

**Affiliations:** a Institute for Cell Biology, University of Bonn, Bonn, 53121, Germany; b NSF Science and Technology Center for Engineering Mechanobiology, Washington University in St. Louis, MO, USA; c Institute of Neuropathology, Justus-Liebig-University Giessen, Giessen, 35392, Germany; d Department of Pathology, University Hospital Bonn, Bonn, 53127, Germany; e Erich and Hanna Klessmann Institute for Cardiovascular Research and Development, Clinic for Thoracic and Cardiovascular Surgery, Heart and Diabetes Center NRW, Bad Oeynhausen, 32545, Germany; f Division of Nephrology, Department of Medicine, Washington University School of Medicine, St. Louis, Missouri, USA

**Keywords:** Synaptopodin, Protein isoforms, Alternative splicing, Filamin, α-actinin, STIM1, Endoplasmic/sarcoplasmic reticulum

## Abstract

Synaptopodin (SYNPO), founding member of the family of podin proteins, was mainly studied in the kidney and the brain. Here, SYNPO was initially described to be expressed in renal podocytes and the endoplasmic reticulum (ER-)related spine apparatus in dendritic spines, respectively. More recently, SYNPO expression was also reported in epithelial cells, and database analyses even indicate ubiquitous expression, including high expression in striated and smooth muscle cells. Our analyses at RNA and protein levels confirm expression of distinct SYNPO variants in all striated and smooth muscle cell-containing organs analyzed and identify isoform-specific expression of SYNPO in human and mouse tissues. Immunostaining of tissue sections revealed diffuse localization at myofibrillar Z-discs in striated muscle fibers and at intercalated discs of cardiomyocytes. In skeletal muscle fibers, but also in cardiomyocytes, SYNPO partially colocalizes with longitudinal sarcoplasmic reticulum (SR-)associated STIM1. In smooth muscle cells, SYNPO shows a reticular distribution, compatible with potential co-distribution with the SR in these cells. In addition to previously reported interactions with actin and α-actinin, our protein interaction studies reveal a small region of SYNPO, homologous to the filamin C (FLNc)-binding region of SYNPO2, to bind FLNc. Transfection of SYNPO isoforms indicate close association with myofibrillar Z-discs in skeletal myotubes as well as stress fiber dense bodies and focal adhesions in smooth muscle cells. Our data identify strong expression of SYNPO variants in all muscle cell types, and indicate a role for SYNPO in the interaction between the actin cytoskeleton and the sarcoplasmic reticulum.

## 1. Introduction

Synaptopodin (SYNPO) is the eponym of the podin protein family, with as further family members synaptopodin-2 (SYNPO2; also known as myopodin or fesselin in birds [1-3]) and synaptopodin 2-like protein (SYNPO2L; also known as tritopodin or CHAP [4,5]). SYNPO is an actin-associated protein that was initially described to be primarily expressed in differentiated podocytes of the kidney, where it appears as punctate structures along actin filaments, and in dendritic spines of cortical and hippocampal neurons [6-8]. In the latter cells, SYNPO is selectively localized to the spine apparatus of dendritic spines, an organelle composed of stacked smooth endoplasmic reticulum (ER) cisternae associated with calcium signaling and synaptic plasticity [9]. Furthermore, SYNPO is a component of the cisternal organelle of the axon initial segment of cortical neurons [10,11]. In the kidney, SYNPO is expressed in podocytes, specialized epithelial cells that enwrap glomerular capillaries and contribute to the integrity of the glomerular filtration barrier. SYNPO localizes to foot processes and regulates actin bundling through interactions with α-actinin, thereby stabilizing the actin cytoskeleton and maintaining filtration slit architecture [12]. It also modulates the activity of Rho-family GTPases, such as RhoA, to control cytoskeletal tension and contraction. Concomitantly, gene silencing of SYNPO in podocytes leads to loss of stress fibers, formation of non-polarized filopodia, and impaired cell migration [13]. Several SYNPO isoforms were reported to directly bind actin filaments and to contain, up to 4 independent α-actinin binding sites [12,14]. Furthermore, SYNPO was shown to act as an upstream regulator of the small GTPase RhoA, whose upregulation induces stress fiber formation [13, 15], highlighting its role in actin dynamics and cell motility.

SYNPO-deficient mice completely lack a spine apparatus in cortical and hippocampal neurons, as well as the cisternal organelle of the axon initial segment of cortical neurons, resulting in impaired intracellular calcium dynamics, disruption of long-term potentiation (LTP), and deficits in learning and memory [9,10,16]. Overexpression of SYNPO in cultured neurons suggests the protein to protect F-actin from degradation, to stabilize activity-dependent increases in the spine apparatus, and to be primarily involved in the late phase of LTP [17]. Additionally, SYNPO was reported to facilitate dendritic spine remodeling following synaptic plasticity-inducing stimulation, which is enabled by the release of Ca^2+^ [18,19]. SYNPO is thus crucial for synaptic plasticity, linking morphological adaptations of the actin cytoskeleton with functional synaptic changes in response to stimulation [16,17]. By contrast, loss of synaptopodin in podocytes only leads to a pronounced phenotype upon injury [12,20]. Upregulation of the isoform SYNPO-T in SYNPO-deficient podocytes but not neurons was suggested to partially compensate the lack of SYNPO in the kidney [12]. This assumption was refuted upon the development of a mouse model with complete absence of all SYNPO isoforms. These mice developed no renal phenotype under physiological conditions [20]. In humans, mutations in the promoter region of the *SYNPO* gene are associated with lower expression of SYNPO in patients with idiopathic focal segmental glomerulosclerosis (FSGS) [21], but consistent with the lack of a phenotype under physiological conditions in *SYNPO* deficient mice, none of the more than 1000 human *SYNPO* variants (https://www.uniprot.org/uniprotkb/Q8N3V7/variant-viewer) was reported to have clinical significance.

Knowledge about the precise gene structure and resulting SYNPO isoforms has been surprisingly sparse. Different isoforms generated through alternative splicing and promoter usage were initially named SYNPO-short (SYNPOb; 685 amino acids in humans, 690 in mice), expressed in the brain, SYNPO-long (SYNPOa; 903 amino acids in humans, 901 in mice) expressed in kidney glomeruli, and SYNPO-T (181 amino acids in mice), found in the kidney of SYNPO knockout mice [12]. Additionally, a 929-amino acid isoform was identified (SYNPOc), which is enriched in epithelial cell-cell junctions [22]. These SYNPO variants consist of a core part encoded by exon 4a, with either no extension (SYNPOb, NP_001103444, UniProt Q8N3V7-3), or with an N-terminal (SYNPOc, NP_001159680, UniProt Q8N3V7-1) or C-terminal extension (SYNPOa, NP_009217, UniProt Q8N3V7-2). Expression of isoforms containing both extensions (SYNPOd, isoform X1, XP_054207515.1, 1147 amino acids) was only appreciated more recently [23]. A schematic representation of the gene structure and the splicing patterns that lead to expression of the different human and mouse *SYNPO* mRNA isoforms is shown in Fig. 1A-C, and the resulting protein isoforms in Fig. 2A).

Apart from its expression in neurons and podocytes, SYNPO was recently reported to be expressed in Madin-Darby Canine Kidney (MDCK) epithelial cells, where it functions as an upstream regulator of α-actinin-4 [22]. SYNPO and α-actinin-4 form a complex together with β-catenin, which coordinates cell-cell adhesion and regulates gene transcription in the Wnt signaling pathway. In addition, SYNPO interacts with myosin II, indicating that SYNPO might act as a link between adhesion molecules and the contractile machinery of the cell. Downregulation of SYNPO resulted in a reduction in α-actinin-4, actin, and vinculin accumulation at cell-cell junctions. Furthermore, wound-induced contraction, which relies on the interplay of actomyosin with cell-cell adhesion, was impaired in SYNPO knockdown epithelial cells suggesting a defect in contractility [22].

The many facets of SYNPO are further exemplified by studies aiming at the identification of SYNPO-binding partners in the spine apparatus by *in vivo* proximity proteomics [24], and by pull-down of SYNPO-associated proteins from rat brain [25] or HeLa cells [26]. In all cases a large variety in interacting proteins was found, that not only include actin and actin-related proteins, but also ER-associated, signaling and scaffold proteins.

Sparse reports indicate that *SYNPO* is also expressed in striated and smooth muscle cells [27-29]. An analysis of *SYNPO* mRNA expression in human tissues by the GTEx portal not only confirms this data, but also indicates that *SYNPO* is almost ubiquitously expressed, and that its expression in smooth as well as striated muscle cells is even higher than in tissues in which expression and function of SYNPO has thus far been mainly studied (Supplementary Fig. 1; https://www.gtexportal.org/home/gene/SYNPO). This prompted us to investigate *SYNPO* mRNA expression as well as protein expression and localization in human and mouse tissues with an emphasis on striated and smooth muscle cells.

## 2. Materials and methods

### 2.1. Human and mouse tissue specimens

Human skeletal muscle samples were collected from the *gastrocnemius* muscle of adult individuals without muscle pathology. Cardiac tissue was obtained from the left ventricle of an explanted heart from a 15-year-old child with acute rejection reaction after heart transplantation at the age of 2 years (immunolocalization assays), or from rejected donor hearts (western blotting). Normal prostate tissue was obtained from the BioMaSoTa-Biobank of the Department for Integrated Oncology (CIO) Bonn. Colon tissue was from a biopsy of a patient with Hirschsprung disease. All material was collected independent of, and prior to our study for diagnostic purposes. Use of the material was approved by the ethical committees of the Universities of Cologne/Bonn (#13-091), the University of Giessen (#AZ07/09), the Ruhr-University Bochum, located in Bad Oeynhausen, Germany (reg.-No. 2013-21, 2017-232-1 and –2) and the ERB of the Medical association Münster/Westphalia, Germany (reg.-No. MS 2017-514-b-S). Written informed consent was obtained from all subjects involved in this study. Mouse tissues were collected from wildtype C57BL/6 mice, that were housed in the animal facility of the University Clinics Bonn following the Federation of European Laboratory Animal Science Associations (FELASA) guidelines. Mice were housed on a 12-h light/dark cycle and had ad libitum access to food and water. A certified veterinarian was assigned to monitor the sanitation of the facility and the health status of the mice.

### 2.2. RT-PCR

For semi-quantitative analysis of SYNPO isoform expression in human and mouse tissues at the RNA level, total RNA was isolated from tissue specimens using the RNeasy Fibrous Tissue Mini Kit following the manufacturer's instructions (Qiagen, Hilden, Germany). cDNA was prepared using random nonamers and the FIREScript RT cDNA Synthesis Kit (Solis Biodyne, Tartu, Estonia) or the Omniscript RT Kit (Qiagen, Hilden, Germany). cDNA was amplified by PCR using the oligonucleotides listed in Table 1 using FIREPol Master Mix Ready to Load (Solis Biodyne) or, in case of high GC amplicons (RT-PCRs involving exon 5), with Phusion High-Fidelity PCR Master Mix with GC-Buffer (Thermo Fisher Scientific, Dreieich, Germany). Oligonucleotides were designed using OligoPerfect Primer Designer (https://www.thermofisher.com/de/de/home/life-science/oligonucleotides-primers-probes-genes/custom-dna-oligos/oligo-design-tools/oligoperfect.html) by submitting the sequence of the regions flanking the individual exon boundaries. Amplicons were analyzed by agarose gel electrophoresis. Gels were photographed using a GelDoc XR Imaging system and Quantity One software (Bio-Rad, Feldkirchen, Germany).

### 2.3. Preparation of tissue extracts and western blotting

Comparative protein expression analyses were performed essentially as described [30]. In brief, frozen tissue samples were weighed, mechanically disrupted using a TissueLyser LT (Qiagen, Hilden, Germany) at 50 Hz, and dissolved in 16 μl urea buffer [2 M thiourea, 7 M urea, 5 mM EDTA, 1 mM DTT and protease inhibitors (Sigma, P8340) in 100 mM Tris pH 8.6] per 1 mg of tissue sample by homogenization for 5 min at 50 Hz. Preheated SDS sample buffer was added to a final 2-fold concentration and samples were incubated for 5 min at 55 °C. For comparative qualitative blotting, similar total protein amounts were separated by SDS polyacrylamide gel electrophoresis (SDS-PAGE) and transferred onto nitrocellulose membranes using a Mighty Small transfer tank (Hoefer, Holliston, MA, USA). Membranes were incubated with primary antibodies and secondary antibodies conjugated with IRDye-800 (LI-COR Biosciences, Bad Homburg, Germany), or horse-radish peroxidase (HRP; Jackson ImmunoResearch). In the latter case, signals were detected using SuperSignal West Pico PLUS Chemiluminescent Substrate (Thermo Fisher). Signals were documented using a ChemiDoc MP Imaging System (Bio-Rad). Further analyses were performed using Image Lab version 6.1.0 software (Bio-Rad).

### 2.4. Immunolocalization in tissue sections

For immunostaining, 6 μm thick cryosections were fixed with acetone (−20 °C) for 10 min and air dried. Subsequently, sections were rehydrated with phosphate-buffered saline (PBS) and incubated with blocking medium (10% normal goat serum in PBS) for 45 min at room temperature (RT). Sections were incubated with primary antibodies diluted in 1% bovine serum albumin (BSA) in PBS overnight at 4 °C. After extensive washing with PBS containing 0.05% Tween 20, sections were incubated at RT with the appropriate secondary antibodies diluted in 1% BSA in PBS for 1 h. Slides were extensively washed with PBS and dipped in water before being mounted in FluoromountG mounting medium (Thermo Fisher). Specimens were analyzed and photographed using a Zeiss LSM900 confocal microscope (Carl Zeiss GmbH, Oberkochen, Germany). For details: see Section 2.12. Controls included staining with secondary antibodies only. No unspecific staining or auto-fluorescence was observed.

### 2.5. Primary and secondary antibodies, and other reagents

Primary and secondary antibodies, and other reagents used for staining in this study are listed in Table 2.

### 2.6. Design of cDNA constructs

Full length cDNAs encoding human SYNPO isoforms b, c and T (UniProt Q9UMS6) were amplified from a cDNA clone (DKFZp451G172Q, obtained from Imagenes, Berlin, Germany), or a construct containing cDNA encoding SYNPOa in a modified pEGFP-C2 vector, that lacked the nucleotides encoding the first 8 amino acids of this isoform (a kind gift of Dr. Joachim Kremerskothen, Münster, Germany). cDNAs were cloned using the appropriate restriction enzymes included in the oligonucleotides used for amplification in a pEGFP-C2 vector with a slightly modified multiple cloning site (Clontech).

### 2.7. Protein expression and purification

Recombinant proteins were expressed in *E. coli* BL21(DE3)Codon Plus cells (Stratagene, LaJolla, CA, USA). Bacteria were grown in LB medium containing 100 mg/l carbenicillin and 34 mg/l chloramphenicol in an incubator shaker at 37 °C. Protein expression was induced at OD600 = 0.6 b y addition of 1 mM IPTG. After 3 h at 37 °C in the shaker, cells were harvested by centrifugation and cell pellets were immediately further treated, or stored at −20 °C until use. Purification of His-tagged proteins was performed essentially as described [3,36]. In brief, cells were lysed in lysis buffer (50 mM sodium phosphate, pH 8.0, 300 mM NaCl, 10 mM imidazole, 1 mg/ml lysozyme), DNA was fragmented by sonification (Hielscher UP 50H, Teltow, Germany), and cell debris was sedimented by centrifugation (4500×*g*, 30 min, 4 °C). Soluble proteins in the supernatant were incubated with Protino Ni-NTA Agarose (Macherey-Nagel, Düren, Germany) under constant agitation at 4 °C for 1 h. Beads were washed twice with washing buffer (50 mM sodium phosphate, pH 8.0, 300 mM NaCl, 20 mM imidazole), and bound protein was eluted with elution buffer (50 mM sodium phosphate, pH 8.0, 300 mM NaCl, 250 mM imidazole), and cOmplete Protease Inhibitor Cocktail (Roche Diagnostics, Mannheim, Germany) was added. Eluted proteins were stored on ice until use.

### 2.8. Co-immunoprecipitation and western blot overlay assays

For co-immunoprecipitation experiments 1 μg T7-tagged and 10 μg EEF-tagged purified recombinant proteins were mixed in IP buffer (0.05% Triton X-100, 1% BSA, protease inhibitors in PBS) and incubated at RT for 1 h. After adding 0.7 μg of the monoclonal antibody (mAb) against the T7-tag, the mixture was further incubated for 30 min at RT. Subsequently, 20 μl Dynabeads protein G (Dynal Biotech, Hamburg, Germany) were added, and the mixture was incubated at 4 °C for 3 h. All previous steps were performed with gentle shaking. The beads were washed three times with IP buffer without BSA and boiled for 3 min in 20 μl SDS sample buffer. Bead-associated proteins were separated by SDS-PAGE, transferred to nitrocellulose membranes and, after blocking in 4 % nonfat dry milk in Tris-buffered saline containing 0.05% Tween (TBST), immunodetected using antibodies directed against the respective tags and HRP-conjugated goat anti-mouse, or goat anti-rat immunoglobulins. Signals were detected using SuperSignal West Pico PLUS Chemiluminescent Substrate, and Super RX-N X-ray film (Fujifilm, Düsseldorf, Germany).

### 2.9. Culture and nucleofection of C2C12 mouse myoblasts

C2C12 myoblasts (RRID:CVCL_0188) obtained from the European Collection of Authenticated Cell Cultures (ECACC) were grown in high glucose Dulbecco's Modified Eagle Medium (DMEM) containing Glutamax, supplemented with 15% fetal calf serum (FCS), 2 mM non-essential amino acids, 100 U/ml penicillin and 100 μg/ml streptomycin (all from Life Technologies, Darmstadt, Germany) to a confluency of approximately 90% at 37 °C and 5% CO_2_ with saturating humidity. Differentiation was induced by changing to the same medium supplemented with 2% horse serum instead of FCS. Differentiation medium was changed every 24–48 h until extensive myotube formation was observed (after 5-6 days).

C2C12 cells were trypsinized and transfected with eGFP-SYNPO-isoform-encoding constructs by nucleofection using a Nucleofector Device, Nucleofector Kit V and program B-032 according to the recommendations of the manufacturer (Lonza, Cologne, Germany). After nucleofection, cells were plated in 35 mm culture dishes and allowed to differentiate. Differentiated cells were fixed in 4% paraformaldehyde, permeabilized using 0.5% Triton X-100, and stained with T12 antibody that recognizes a titin epitope close to the Z-disc and the appropriate secondary antibody (GAM IgG1 AlexaFluor594). Cells were analyzed with an LSM900 confocal laser scanning microscope (Carl Zeiss).

### 2.10. Electrical pulse stimulation

In order to enhance differentiation and induce myofibrillar lesions, C2C12 myotubes were treated mainly as described [37]. In brief, cells were grown in 6 well plates for western blotting experiments or isolation of RNA, and on glass coverslips placed in 6 well plates for microscopical analyses, or in 35 mm culture dishes with cover-glass bottom (Fluorodish, World Precision Instruments, Sarasota, FL, USA) for live-cell imaging. Cells were electrically stimulated by applying pulses of 10 V for 10 ms at a frequency of 1 Hz using a C-Pace EP culture pacer (Ion Optix, Milton, MA, USA) and a six-well C-dish (Ion Optix).

### 2.11. Culture and transfection of A7r5 smooth muscle cells

A7r5 smooth muscle cells (RRID:CVCL_0137) were originally obtained from the American Type Culture Collection (ATCC CRL-1444), and kindly provided by Dr. Mario Gimona (Salzburg, Austria). Cells were grown in low glucose (1 g/l) DMEM without phenol red containing 10% FCS (Sigma), 4 mM L-glutamine, 100 U/ml penicillin, 100 μg/ml streptomycin (all from Gibco/Thermo Fisher) at 37 °C and 5% CO_2_ with saturating humidity. Cells were transfected with a plasmid encoding T7-tagged C-terminal half of human SYNPOb (H1-H3; amino acids 348-685) or a fusion protein of eGFP and different SYNPO isoforms using Jet-PRIME transfection reagent (Polyplus, Illkirch, France), following the instructions of the manufacturer. 48 h after transfection, cells were fixed for 10 min with a 1:1 mixture of methanol and acetone at −20 °C (T7-tagged protein) and stained with antibodies against the T7-tag and filamin. Cells expressing eGFP-fusion proteins were fixed with 4% PFA, permeabilized with 0.5% Triton X-100, and stained with B-12, an antibody specific for α-actinin, and the appropriate secondary antibody (GAM IgG2a Alexa 594), while the actin cytoskeleton was stained using PromoFluor-504-phalloidin (PromoKine, Heidelberg, Germany). Controls included staining with secondary antibodies only. No unspecific staining was observed.

### 2.12. Confocal laser scanning microscopy

For visualization of fluorescence-labelled cells and tissue sections, a confocal laser scanning microscope was employed (LSM 900 Airy Scan, Carl Zeiss) using an EC Plan-Apochromat DICII 40x/1.3 oil objective. The diameter of the pinhole was set to one airy unit (AU) for all wavelengths. To prevent signal crosstalk when imaging, multi-channel labelling, line-by-line scanning was performed recording each channel separately and sequentially. The mean value of four recordings was formed for each pixel, with the pixel dwell time set as high as possible. Digital image acquisition and processing was performed using ZEN blue software Version 3.5 (Carl Zeiss). Representative images were chosen to prepare figures. Figures were generated using CorelDRAW Version 26.0.0.101 (Corel Corporation, Ottawa, Canada).

### 2.13. Modeling of protein-protein interactions

Protein–protein interaction models were generated using AlphaFold Server (powered by AlphaFold 3; https://alphafoldserver.com/) [38]. Amino acid sequences of the two proteins portions to be analyzed were provided as input, and complex predictions were performed using default parameters. The highest-confidence model was selected for downstream analyses. Model confidence was evaluated using the per-residue predicted Local Distance Difference Test (pLDDT) scores and predicted aligned error (PAE) metrics provided in the output files. Interface confidence and relative domain positioning were assessed based on PAE values. Structural coordinates and associated JSON output files were downloaded and further analyzed using the PyMOL Molecular Graphics System, Version 3.0 Schrödinger, LLC. Interface contacts and structural representations were generated within PyMOL.

## 3. Results

### 3.1. SYNPO is highly expressed in striated muscles and smooth muscle tissue containing organs

Since there were clear indications that SYNPO is not only expressed in kidney and brain but also in other tissues (Supplementary Fig. S1 [27, 28]), we analyzed the expression of SYNPO in human and mouse tissues, with an emphasis on striated and smooth muscle cell-containing tissues, at the mRNA level using RT-PCR and exon-specific primers. Isoform-specific RT-PCRs indeed revealed clear differences in the expression patterns in human tissues: while in the heart the strongest signals were obtained using the primer pairs exons 3-4a and exon 4a-4b, indicating mainly expression of SYNPOb, in the prostate the primer pairs exons 4a-5 resulted in a relatively strong signal, implying expression of isoform SYNPOa (Fig. 1B). Comparison of this data with analyses by the Human Protein Atlas (HPA, https://www.proteinatlas.org/) and GTEx essentially confirm our results (Fig. 1B, Supplementary Fig. S2).

Similar experiments using RNA purified from different mouse tissues containing either cross-striated or smooth muscle cells revealed expression of *SYNPO* in all analyzed specimens. In the heart all primer combinations except 4c-5 (SYNPO-T) resulted in an amplicon, meaning that RNA variants containing the 5′ extension (exons 1-4a) as well the 3′ extension are expressed. By contrast, in skeletal muscle (*M. gastrocnemius*) no RNA with the 5′ extension and only minimal amounts of the 3′ extension were found. Interestingly, the diaphragm, which can be considered a specialized skeletal muscle, showed an expression pattern highly similar to that of the heart. In bladder and kidney, a stronger signal was obtained with primer pair exons 4a-5 when compared to pair exon 4a-4c, indicating mainly expression of an isoform containing the 3′ extension. The mouse prostate seems to express only very low *SYNPO* levels: only primer pair 4a-4b resulted in a weak signal (Fig. 1D). Differences between human and mouse tissues are probably due to the distinct physiology of both species. In this respect, it is interesting to note that SYNPO expression levels in some human and mouse tissues differ considerably. A comparison of expression levels at BioProject (mouse: PRJNA66167; human: PRJEB4337) indicates an approximately 6 to 8 times higher expression in the human heart and liver, respectively, and an approximately 4 to 7 times lower expression in the human duodenum and ovary, respectively [39,40].

The above results indicated the expression of different splice variants in all muscle cells-containing tissues, prompting us to analyze SYNPO protein expression using two different well-characterized SYNPO-specific antibodies, both recognizing all SYNPO isoforms except SYNPO-T. In human cardiac and skeletal muscle the G1D4 mouse monoclonal antibody (which binds only a single epitope) detected an approximately 110 kDa protein in western blots of both striated muscle types, but no signal in smooth muscle cells-containing human prostate extracts (Fig. 2B, left panel). A polyclonal rabbit antiserum, raised against a recombinant protein representing aa 135-256 of mouse SYNPOb (142-264 of human SYNPOb) that most probably binds multiple epitopes and thus is more sensitive, stained a similar band in extracts from human striated muscles. In human cardiac tissue extracts, this serum detected two much weaker additional bands of approximately 130 and 150 kDa. These bands were also weakly stained in human prostate extracts, while in mouse uterus extract a protein of ~140 kDa was detected (Fig. 2B, right panel). A faster migrating band running at ~90 kDa in the human heart sample is probably the result of proteolysis. Subsequently, we compared the expression levels of SYNPO in different mouse muscles. In all striated muscles a protein of ~115 kDa was stained, in the uterus low levels of a ~140 kDa protein, and in aorta, in humans the tissue with the highest expression (Supplementary Fig. S1), three bands were detected with an apparent molecular mass of 140, 160 and 200 kDa, respectively (Fig. 2C). A comparison of our western blotting data with mRNA expression data obtained from HPA and GTEx, mainly confirm our observation (Supplementary Fig. S3).

### 3.2. SYNPO is associated with Z-discs and the sarcoplasmic reticulum in skeletal muscle fibers

To analyze the localization of SYNPO in skeletal muscle, cryosections of human skeletal muscle specimens were stained with anti-SYNPO antibodies in combination with antibodies specific for Z-disc-associated proteins α-actinin and desmin. This indicated that SYNPO is localized in a cross striated pattern which is at low magnification similar to the localization of both α-actinin and desmin (Fig. 3A). While at higher magnification, α-actinin, as component of the central Z-disc, and desmin, a component of the intermyofibrillar cytoskeleton, are clearly separated (Fig. 3A'), SYNPO does not completely colocalize with either α-actinin or desmin.

To better identify the localization of SYNPO, double stainings were performed with antibodies against the ryanodine receptor (RyR) and STIM1, both proteins reported to be associated with SYNPO in other cells [16,19]. Here, SYNPO did not show any colocalization with RyR that in skeletal muscle fibers is localized in the triads close to the junction between I-bands and A-bands (A/I junction) (Fig. 4A and A'). In contrast, partial colocalization of SYNPO with STIM1, a protein associated with the endoplasmic/sarcoplasmic reticulum (ER/SR), and with Z-disc protein and SYNPO-binding partner FLNc (see section 3.5) was observed (Fig. 4B).

### 3.3. SYNPO is associated with intercalated discs and Z-discs in the human heart

Immunolocalization of SYNPO together with SYNPO2 and FLNc in cryosections of a human heart specimen revealed that all three proteins are localized at Z-discs (Fig. 5A). The major portion of FLNc is, however, associated with intercalated discs (IDs). The low exposure times needed to avoid overexposure of IDs therefore resulted in a rather low signal in Z-discs. While SYNPO2 was excluded from IDs, both SYNPO and FLNc were found in IDs, however in distinct locations: FLNc showed a mainly filamentous staining pattern in IDs, whereas SYNPO distribution was more diffuse and therefore only partially overlapping with the FLNc signal (Fig. 5A').

Staining of these sections for STIM1 in combination with SYNPO and FLNc indicated only very limited localization of STIM1 in cardiac Z-discs, and no accumulation of STIM1 in IDs (Fig. 5B). The more diffuse, reticular distribution of STIM1, resulted in a considerable colocalization of SYNPO with STIM1, but apart from the limited localization of STIM1 in Z-disc areas, significant concentration of STIM1 in SYNPO-containing structures was not observed.

### 3.4. SYNPO is distributed in a reticular pattern in human colon smooth muscle cells

Data from the GTEx portal and HPA indicated that SYNPO is expressed in smooth muscle cell-containing tissues (Supplementary Fig. S1), which is confirmed by single cell analyses by HPA. The relative level of SYNPO mRNA varies considerably between 10.5 and 181.5 transcripts per million (TPM) in endometrial and vascular smooth muscle cells, respectively (Supplementary Table 1). To analyze the distribution of SYNPO in smooth muscle cells, we stained cryosections of human colon with antibodies specific for SYNPO, SYNPO2 and FLN. In cross sectioned cells, both FLN and SYNPO2 showed a distribution in discrete, but mostly non-overlapping spots. In contrast, SYNPO was not localized to these structures, but rather in a reticular pattern, that only partially overlapped with the SYNPO2 and FLN localizations (Fig. 6A). In longitudinally sectioned smooth muscle cells, the reticular distribution of SYNPO was confirmed (Fig. 6B). Also here, partial colocalization of SYNPO with FLN-containing filaments or SYNPO2-containing dense bodies was observed (Fig. 6B’). In the colon, SYNPO was also detected mainly at the apical site of the epithelial cells of the intestinal glands (Supplementary Fig. S4).

### 3.5. SYNPO directly interacts with FLNc

SYNPO contains three homology regions (H1-H3) that all proteins of the podin family have in common (Fig. 2A). For SYNPO2 this part of the protein (more precisely: H1-H2) was shown to interact with FLNc [3]. To analyze whether the corresponding portion of SYNPO has similar properties, we started with building a construct encompassing this core region (SYNPO H1-H3; SYNPOb amino acids 348-685) fused to a T7-tag. Expression of this fusion protein in A7r5 smooth muscle cells resulted in targeting to stress fiber-associated dense bodies and adhesion plaques that were identified by co-staining for FLN (Fig. 7A). Since such a localization is in line with the above mentioned SYNPO2 - FLNc interaction, we used the AlphaFold Server (powered by AlphaFold 3; https://alphafoldserver.com/) to search for potential interaction sites between SYNPO (H1-H3) and FLNc (Ig-like domains d20 and d21). An initial analysis using SYNPO H1-H3 and FLNc d20-21 predicted with high confidence a site in human SYNPO H2 to interact with FLNc d21. Further analysis performed with FLNc d21 only and the interacting region of SYNPOa GKGAELYARRQS_489_RME_492_K_493_Y_494_VI_496_ESSSHT, revealed interaction of the underlined amino acids with the underlined amino acids in peptide AG_2345_GL_2347_SI_2349_AVE_2352_G representing β-strand C of FLNc d21. Furthermore, AlphaFold predicted an interaction between SYNPO K_493_ and FLNc D_2389_ (loop between β-strands F and G), and between SYNPO M_491_ and FLNc K_2386_ (β-strand F) but analysis of this data by PyMol (Fig. 7B) revealed that the distance between the donor and acceptor atoms of the latter interacting amino acids exceeded the cutoff of 3.5 Å applied here. This cutoff determines when a contact between label and protein is counted as a clash and is a typical distance between residues in the hydrophobic core of a protein structure [41]. An interaction of the above mentioned SYNPO peptide was also predicted with FLNc d17 and FLNc d19, although with much lower confidence (not shown). No confident interaction was predicted upon modeling of SYNPO H1 or SYNPO H3 with FLNc d21 using AlphaFold.

To confirm this prediction, we performed co-immunoprecipitation assays using recombinantly expressed SYNPO fragments (for solubility reasons we had to use the entire H1-H3 portion or an H2-H3 fragment) and the FLNc subfragment d18-21. Indeed both SYNPO fragments (H1-H3 and H2-H3; SYNPO2b amino acids 348-685 and amino acids 452-685, respectively) co-immunoprecipitated with FLNc d18-21, indicating a direct interaction of SYNPO H2-H3 with FLNc (Fig. 7C and D). Similarly, the subfragment FLNc d20-21 was also precipitated together with SYNPO H2-H3 (Fig. 7E).

Taken together, the homology between SYNPO and the interacting region in SYNPO2 (H1-H2), and the above described transfection, modeling and biochemical data have allowed to reveal a direct interaction of a conserved peptide in the H2 region of SYNPO and FLNc d21.

### 3.6. All SYNPO isoforms are targeted to Z-discs upon expression in mouse myotubes

Since the applied antibodies neither detected SYNPO protein in cultured C2C12 myotubes by western blotting, nor by immunofluorescence staining (results not shown), we transfected C2C12 myoblasts with constructs encoding eGFP fusion proteins of SYNPOa, b, c and SYNPO-T by nucleofection. All variants were at least in part targeted to myofibrillar Z-discs that were identified by co-staining for a titin epitope close to the Z-disc (Fig. 8A-D). Isoforms SYNPOa, b and c were in addition found in vesicular structures not associated with Z-discs. Isoform T was not targeted to such structures but instead showed a more diffuse distribution. Electrical pulse stimulation (EPS) of the transfected myotubes was applied to stimulate contraction of myotubes, either to promote sarcomere assembly, or to simulate exercise leading to the induction of sarcomeric damage. This resulted in a more pronounced localization of isoforms SYNPOa, b and c to Z-discs (Fig. 8A-D). Furthermore, all SYNPO isoforms were found in small longitudinal structures that were not stained by the anti-titin antibody and most likely are microlesions since they have the typical appearance of these EPS-induced structures [37,42,43].

Transient expression of the eGFP-SYNPO isoforms in A7r5 smooth muscle cells resulted in targeting of all isoforms to stress fiber dense bodies that in some cases were aligned and appeared as sarcomere-like structures (Fig. 9A-D). Interestingly, and in contrast to SYNPOb and c, SYNPOa and SYNPO-T were also localized in adhesion plaques at the end of the stress fibers (Fig. 9A-D).

## 4. Discussion

Since its discovery in renal podocytes and dendritic spines of the spine apparatus in the brain [6], synaptopodin (SYNPO) was mainly studied in these structures. Despite its robust expression in striated as well as smooth muscle cells, expression of SYNPO in these tissues was, although noted [27-29], never analyzed in detail. Together with data obtained from GTEx portal and HPA, our RT-PCR, western blot and immunolocalization studies presented in this work not only confirm the expression of SYNPO in these tissues, but in addition, they also reveal its almost ubiquitous expression across tissues. Distinct SYNPO isoform profiles indicate tissue-specific mechanisms that regulate isoform expression, probably linked to a functional specialization of the respective SYNPO isoforms. In this respect, it should also be noted that the PhosphositePlus database (https://www.phosphosite.org/) lists 30 different phosphorylation sites with 5 or more references in mouse and human SYNPO, and more than 80 different sites with at least 1 reference, while in the murine glomerulus, SYNPO is one of the most phosphorylated proteins with 17 phosphorylation sites [44]. Individual phosphorylation sites can be dephosphorylated by calcineurin, thereby preventing binding of 14-3-3 protein family members, which function as chaperone-like phospho-serine/threonine-binding proteins. This dephosphorylation, which can be inhibited by cyclosporin A, facilitates degradation of SYNPO by cathepsin L. This mechanism regulates the stability of the actin cytoskeleton in podocytes [15].

SYNPO mRNA isoforms mainly differ by the usage of alternative transcription start points and inclusion or exclusion of exons by alternative splicing, resulting in the expression of the shortest isoform SYNPOb (also called SYNPO short), or of longer isoforms due to the addition of N-terminal and/or C-terminal extensions (see Figs. 1 and 2). SYNPOa (also called SYNPO long) contains a highly proline-rich 223 or 230 amino acids long extension (mouse and human, respectively) that not only contains two independent α-actinin binding sites [12], but also LPPPP and PPXXF motifs that are known binding sites for EVH1 domains, found in proteins like Ena, VASP and HOMER. Upstream exons (SYNPOc and d) encode a further LPPPP/FPPPP (human and mouse, respectively) motif. The existence of several isoforms with both extensions was predicted (RefSeq database: transcripts with different 5′ predicted exons all encoding isoform X1 with a length of 1147 amino acids and a predicted mass of 122 kDa [https://www.ncbi.nlm.nih.gov/datasets/gene/id/11346/products/]; mouse SYNPO variants X1–X4), and indications for its expression in podocytes and T84 intestinal cells were found [23]. Interestingly, our immunolocalization data are in agreement with the apical localization of SYNPO in monolayers of cultured T84 cells: staining of SYNPO in human colon confirmed the apical localization in the epithelial cells of the intestinal glands of a not yet identified isoform in enterocytes. Unfortunately, isoforms containing exons encoding both, the N-terminal and the C-terminal extension were not considered by the GTEx portal, but in many tissues the exons encoding the N-terminal extension as well the C-terminal extension are expressed, which might indicate expression of isoform SYNPOd. Also our RT-PCR data on mouse tissues revealed the expression of both, the N-terminal as well as the C-terminal expression in e.g. mouse kidney, urinary bladder and the heart. However, these experiments cannot discern between the expression of SYNPOa, c and d.

In skeletal muscle, SYNPO localizes to the Z-disc region, only partially overlapping with desmin and α-actinin, but also showing a unique distribution different from the central Z-disc proteins α-actinin and SYNPO2. This suggests a specific structural or regulatory function distinct from both Z-disc proteins. Indications that SYNPO might be associated with the ER/SR and that SYNPO recruits STIM1 to the post-synaptic density [19,45] prompted us to compare the localization of STIM1 with that of SYNPO also in muscle cells. Indeed, in all muscle types at least partial colocalization of SYNPO with STIM1 was observed. STIM1 is an ER/SR calcium sensor that mediates store-operated calcium entry (SOCE) via interaction with Orai1 channels following store depletion. In skeletal muscle fibers, where STIM1 is mainly localized in the longitudinal SR between triads, the colocalization was most prominent. Since SYNPO can at the same time interact with actin-associated scaffold proteins and cytoskeletal adapters like α-actinin and FLNc, SYNPO may thus participate in anchoring or spatially organizing STIM1-positive ER/SR domains, especially near contractile or mechanosensitive regions. This could serve to stabilize ER/SR-plasma membrane junctions, facilitate SOCE channel clustering, and/or regulate cytoskeleton-dependent calcium microdomains during muscle contraction or contractile stress. The necessary remodeling or repair mechanisms require both coordinated calcium handling and cytoskeletal adaptation. Interestingly, especially in exercised muscle fibers, extensive remodeling of the triads occurs, and Ca^2+^ Entry Units (CEUs) assemble, which are SR-stacks, similar to those observed in dendritic spines, associated with elongated T-tubules. They are formed between existing triads localized at the sarcomeric A/I junction, i.e. in the Z-disc region close to the localization of SYNPO [46,47]. Thus, SYNPO may represent a link between mechanotransduction and calcium entry pathways.

Interestingly, SOCE also seems to play an important role in maintaining the structural and functional integrity of podocytes [48]. Although there is no evidence that SYNPO is an ER-associated protein in podocytes, in these cells pharmacologic ER stress and insulin-induced activation of SOCE are associated with decreased SYNPO levels [49, 50]. In the mouse heart, in turn, STIM1 expression is increased upon transaortic constriction-induced hypertrophy, associated with an increase of SOCE [51], supporting the idea that STIM1-mediated SOCE is associated with hypertrophy [52]. In smooth muscle cells SOCE is mainly active in non-contractile, proliferative cells, and it is upregulated during hypertension [53]. This indicates that in striated as well as smooth muscle cells different types of stress regulate SOCE-activity.

In cardiac tissue, SYNPO is associated with both, Z-discs and intercalated discs (IDs), but unlike FLNc (Z-discs and IDs) and SYNPO2 (Z-discs), SYNPO shows a more diffuse staining pattern, possibly indicating a role in signaling rather than mainly structural integrity. The dual localization might represent co-expression of different isoforms in the heart (Fig. 2B, https://gtexportal.org/home/gene/SYNPO#gene-transcript-browser-block). Using proximity biotinylation assays SYNPO was recently found in the primary neonatal mouse cardiomyocyte inter-actomes of the desmosomal protein desmoglein 2 (DSG2), as well as N-cadherin (CDH2), a cardiac adherens junction protein [54,55]. It is tempting to speculate that in IDs, which are cell-cell contact sites, a different, longer isoform is expressed than in the Z-disc region. This would be an isoform including one or more EVH1-binding sites capable of interacting with Enah and/or VASP, both EVH1 domain-containing proteins that are, like DSG2 and CDH2, associated with the ID [56]. The expression of different SYNPO isoforms in the heart could also result from different cell types that express specific SYNPO isoforms, including cardiomyocytes, smooth muscle cells, endothelial cells and fibroblasts (https://www.proteinatlas.org/ENSG00000171992-SYNPO/single+cell/heart+muscle). The precise identification of the localization of different isoforms must await the development of reliable isoform-specific antibodies.

The expression level of SYNPO in smooth muscle cells is highly variable and differs from 10.5 to 181.5 normalized TPM (nTPM) in smooth muscle cells of the endometrium and vascular smooth muscle cells, respectively (https://www.proteinatlas.org/ENSG00000171992-SYNPO/single+cell; Supplementary Table 1). In mouse aorta we detected three different SYNPO variants, all larger than SYNPOb, which in turn is mainly expressed in cardiac and skeletal muscles (Fig. 2C). This might be the result of post-translational modifications (PTMs), but it is more likely that isoforms SYNPOa, c and/or d are co-expressed. According to single cell analyses by the HPA (https://www.proteinatlas.org/ENSG00000171992-SYNPO/single+cell), in blood vessels SYNPO is expressed in several, very different cell types such as endothelial cells (72.8 nTPM), smooth muscle cells (181.5 nTPM), fibroblasts (34.7 nTPM) and Schwann cells (26.1 nTPM), which might explain the presence of different isoforms or differently modified proteins in the total extract of the aorta. In smooth muscle cells of the human colon, SYNPO displays a reticular distribution, partially colocalizing with FLN and SYNPO2, supporting a scaffolding function, likely adapted to the smooth muscle cytoskeletal architecture. This localization reflects the organization of the SR, which is rather different in the three types of muscle cells. In smooth muscle cells, the ER is irregularly localized in a reticular distribution, without distinctive organization [57,58], contrasting the highly organized situation in skeletal muscle fibers. In these cells it forms triads together with the T-tubules at the level of the junction between the A-band and the I-band, whereas in cardiomyocytes the SR is less well developed. Here, one small terminal cistern forms a diad with a T-tubule at the level of the Z-disc.

Indications that SYNPO might be associated with the ER were recently strengthened by two studies [24,59]. SYNPO-BioID2, when expressed in cultured neurons, is targeted to dendritic spines. In addition, an *in vivo* proximity proteomics assay revealed that expression of the same construct in the cerebral cortex of mice resulted in biotinylation of a large set of proteins including not only actin-related proteins, but also signaling and ER-associated proteins [24]. In a later study the same authors showed that expression of ER-targeted SYNPO in non-neural cells is sufficient to induce the assembly of stacks of ER-sheets that resemble the spine apparatus in dendritic spines, suggesting that SYNPO crosslinks the ER to actin filaments [59].

The targeting to Z-discs of SYNPO isoforms upon transient expression in C2C12 mouse myotubes is compatible with the α-actinin-binding sites present in all isoforms. Isoforms SYNPOa, b and c also localized to vesicular structures, suggesting additional functions beyond sarcomeric integration. The more diffuse distribution of SYNPO-T potentially reflects its truncated structure and associated lack of part of the α-actinin-binding sites [12] and the FLNc-binding site identified in this work. In addition it seems to lack yet unidentified vesicle-targeting motifs. All SYNPO isoforms except SYNPO-T contain PPxY motifs capable of binding WW domains of not only MAGI-1 [60] but also the co-chaperone BAG3 [61]. The interaction between BAG3 and SYNPO2 is important for chaperone-assisted selective autophagy (CASA) in several cell types, and in neurons, the interaction between BAG3 and SYNPO is important for the autophagic clearance of specific proteins [61], indicating that SYNPO might have a similar function in other tissues. The localization of SYNPO in microlesions, structures in which extensive remodeling takes place [30,42,62], points to a role for SYNPO in clearance of damaged proteins.

Transfection of SYNPO isoforms in A7r5 smooth muscle cells resulted in targeting of all isoforms to stress fiber dense bodies, while SYNPOa and SYNPO-T were also localized in adhesion plaques, indicating a potential function of the LPPPP and PPXXF motifs in this part of the protein. Upon expression of mRFP-tagged SYNPO in COS-7 cells, SYNPO localizes to F-actin-containing structures [24]. Depolymerization of actin filaments by treatment with Latrunculin A, however, resulted in the collapse of SYNPO onto the ER, again indicating a close association of SYNPO with the ER/SR [24]. In line with this, in tissue sections, SYNPO shows a distribution in smooth muscle cells that is not clearly associated with actin filaments.

It must be noted, that SYNPO protein is not expressed at detectable levels in early cultured myotubes, indicating that SYNPO does not seem to have an important function in myofibril assembly. This observation is supported by the fact that SYNPO deficiency does not have a major impact on myogenesis or muscle function in SYNPO knockout mice, since a major phenotype would certainly have been reported [9,10,20]. Interestingly, the development of T-tubules and the reorganization of the SR are also only detected in terminally differentiated myotubes. At early stages the distribution of T-tubule and SR-components that are later in development concentrated in triads accumulate in clusters with a vesicle-like distribution [63-65] similar to that of SYNPOa, b and c upon expression in myotubes. During skeletal muscle differentiation, the SR is organized in specific domains in a stepwise process. In adult muscle fibers the SR is highly organized in a junctional SR in triads, with its calcium release channels, and in a longitudinal SR, ensheathing the complete myofibril, where calcium reuptake takes place. The specific functions of the SR domains necessitate individual binding partners specifying the organization of its sub-compartments.

Among the SYNPO-interacting proteins in the spine apparatus of dendritic spines, identified by a proximity biotinylation approach, were PDLIM1 and PDLIM7, and further strong indications for a direct or indirect interaction of SYNPO and PDLIM7 were presented [24]. Using AlphaFold2 the three LIM domains of PDLIM7 were confidently predicted to interact with several divergent SYNPO sequences [66]. Considering the high homology of the LIM domains of the PDLIM family of proteins, SYNPO might as well interact with the LIM domains of PDLIM1/CLP-36, a protein that not only binds the Z-disc proteins α-actinin, myotilin and the calsarcin/myozenin/FATZ family proteins, but also STIM1 [67]. It is therefore plausible to assume that these sequence elements help to link SYNPO to actin filaments and create a molecular matrix for ER/SR (re) organization not only in the spine apparatus, but also in muscle cells. Another interesting potential SYNPO binding partner identified in interaction assays is plectin [24,25], a specific isoform of which links the desmin intermediate filament system to the SR [68]. In addition, desmin was reported to bind STIM1 and to help regulate STIM1 localization at the level of the Z-disc, and thus STIM-activated Ca^2+^ signals in skeletal muscle fibers [69]. Further investigations are necessary to identify the precise role of SYNPO in this complex.

An important question is why cells apparently express different podin proteins simultaneously. While the main homology regions H1-H3 seem to be responsible for binding to α-actinins and filamins, the limited homology of the rest of the proteins therefore must be the main determinants of the divergent functions of the individual isoforms. The presence or absence of PDZ domains, PPxY motifs and EVH1 domain binding motifs most likely play important roles in this context. Here our data provide further indications for a unique scaffolding and/or regulatory role for SYNPO in defining ER/SR architecture, which is particularly intriguing in the context of contractile stress and remodeling, where calcium handling and cytoskeletal adaptation are coordinated. SYNPO may therefore represent a novel and underappreciated link between mechanotransduction and calcium entry pathways.

## Supplementary Material

1

## Figures and Tables

**Fig. 1.**
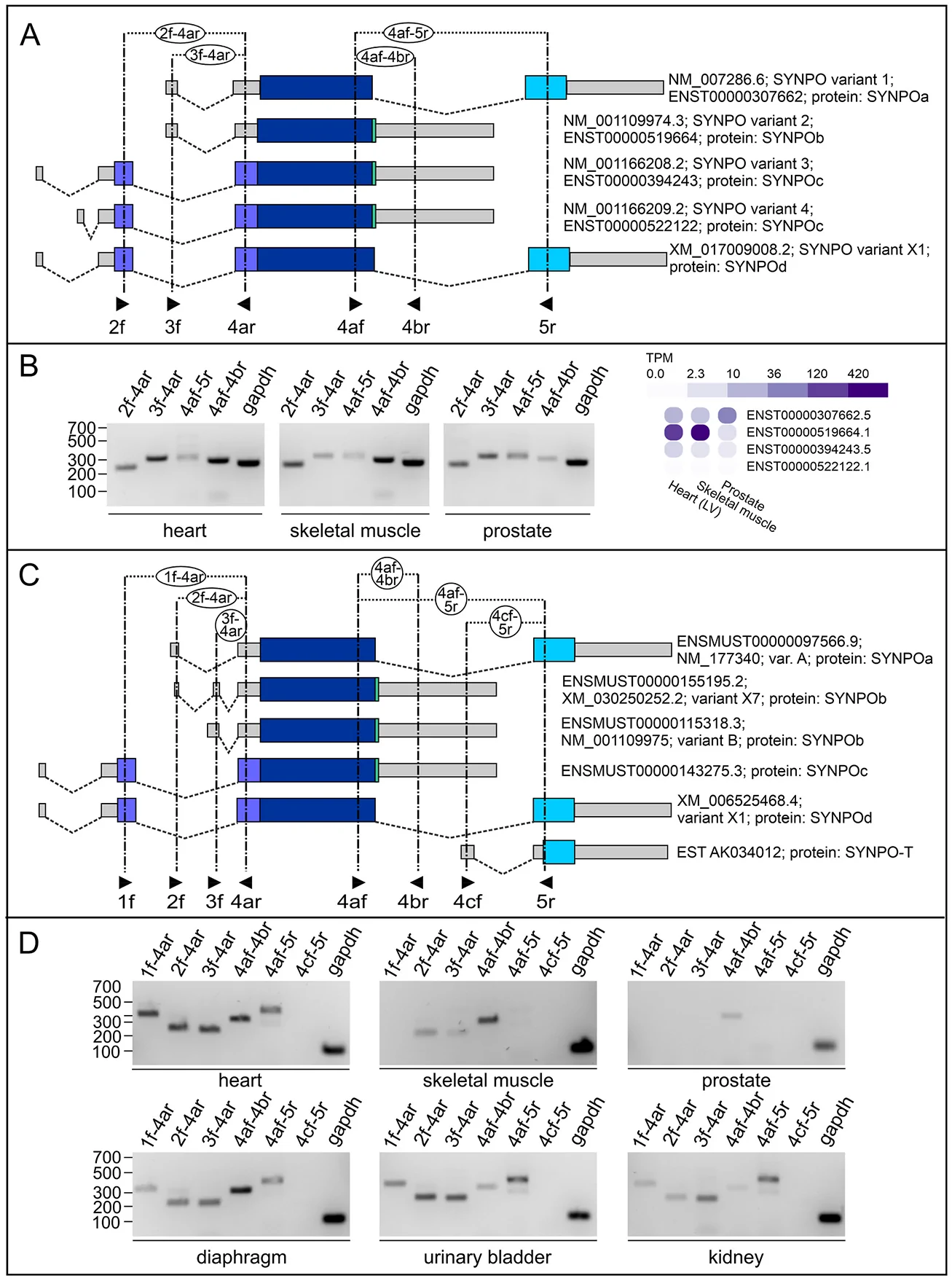
Analysis of SYNPO isoform expression in human and mouse tissues. (A) Schematic presentation of the structure of the human SYNPO gene and splice patterns. Nomenclature of the variants is given at the right. Curated NCBI RefSec variants 1–4 and the predicted variant X1 are alternatively spliced. The color of the exons was chosen to explain which exon encodes specific parts of the SYNPO protein variants depicted in Fig. 2A. (B) Analysis of SYNPO expression at the RNA level in different human tissues by RT-PCR. Positions of primer pairs are indicated in (A). Primers specific for the human housekeeping gene glyceraldehyde-3-phosphate dehydrogenase (G*APDH)* were used as control. The right panel gives data on SYNPO isoform expression in human tissues according to GTEx. (C) Schematic presentation of the structure of the mouse SYNPO gene and splice patterns. Depicted are curated (variants A and B) and a selection of predicted NCBI RefSec variants. Nomenclature of the variants is given at the right. (D) Analysis of SYNPO expression at the RNA level in different mouse tissues by RT-PCR. Positions of primer pairs are indicated in (C). Primers specific for the mouse housekeeping gene *Gapdh* were used as control.

**Fig. 2.**
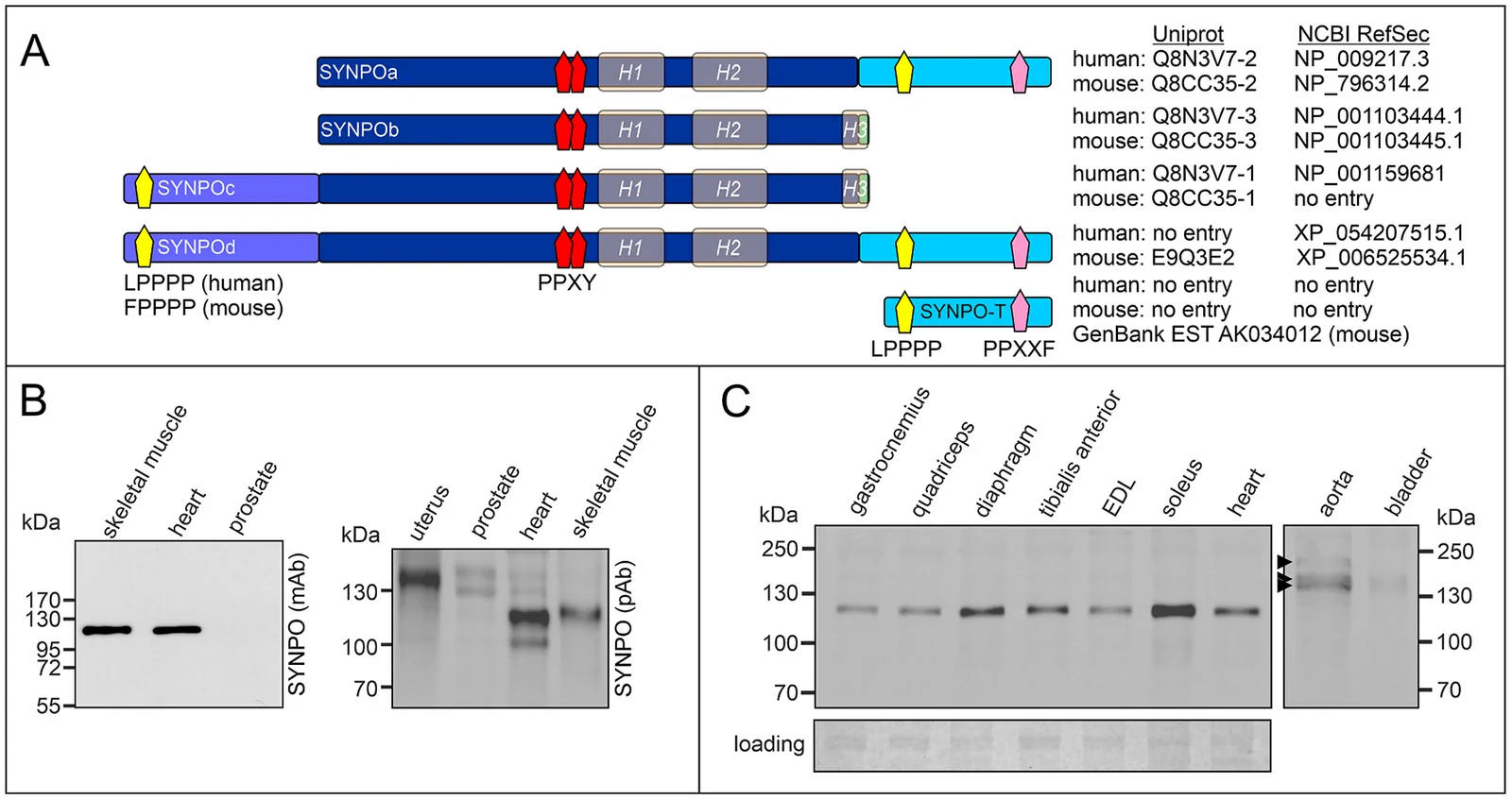
SYNPO isoforms and their expression in human and mouse tissues. (A) Schematic structure of different human and mouse SYNPO isoforms. *: isoform d represents isoform X1 presented in Ref. [23], and summary of the nomenclature of the human and mouse isoforms by UniProt and NCBI RefSec. Different protein regions are colored according to the colors of the respective encoding exons depicted in Fig. 1A. (B) Analysis of SYNPO expression by western blotting confirms the expression of different isoforms in different human (prostate, heart, skeletal muscle) or mouse (uterus) muscle types and tissues. Similar protein amounts were loaded. Blots were either stained with a mouse monoclonal (mAb) or a rabbit polyclonal (pAb) anti-SYNPO antibody recognizing all SYNPO isoforms, as indicated. Note that probably due to the high proline content of all isoforms, the proteins migrate slower than expected. (C) Expression of SYNPO in different mouse striated muscles and smooth muscle-containing tissues. All striated muscles seem to express different levels of the same isoform, probably SYNPOb. In mouse aorta three different isoforms (possibly SYNPOa, c and d) are detected (arrowheads) and in bladder a similar pattern is observed at far lower levels and no detectable expression of the upper band. EDL: extensor digitorum longus.

**Fig. 3.**
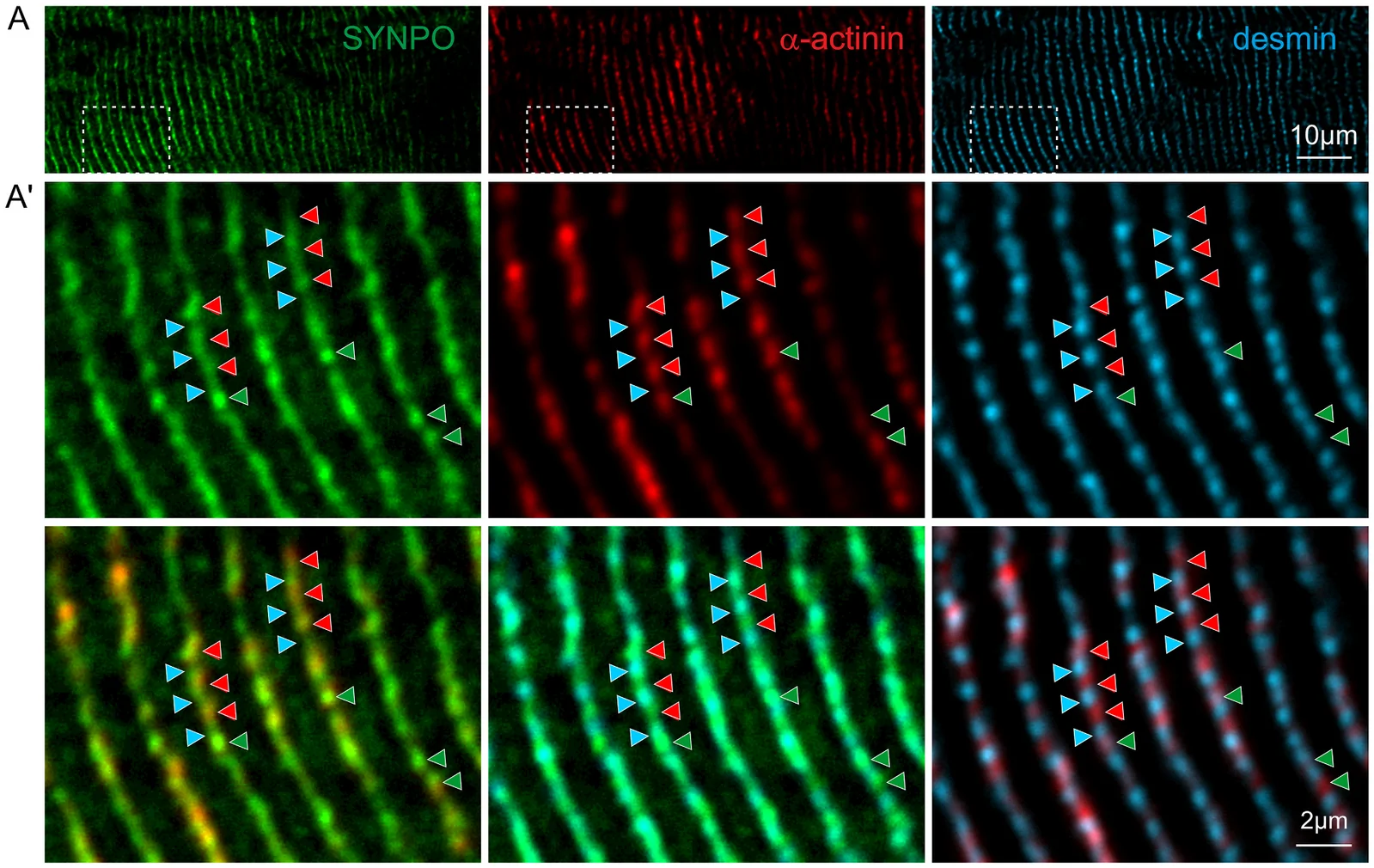
Immunofluorescence localization of SYNPO, α-actinin and desmin in human skeletal muscle fibers. (A) Immunolocalization of SYNPO, α-actinin and desmin in cryosections of human skeletal muscle. All proteins are associated with myofibrillar Z-discs that were identified by staining of the central Z-disc component α-actinin. (A′) Higher magnification of the boxed area in (A) reveals a separation of the desmin (blue) and α-actinin (red) signals. SYNPO distribution is less well defined, and a specific colocalization with either α-actinin or desmin is not observed. For clarity single stainings (A, A′ upper panel) and double stainings (A′ lower panel) are shown.

**Fig. 4.**
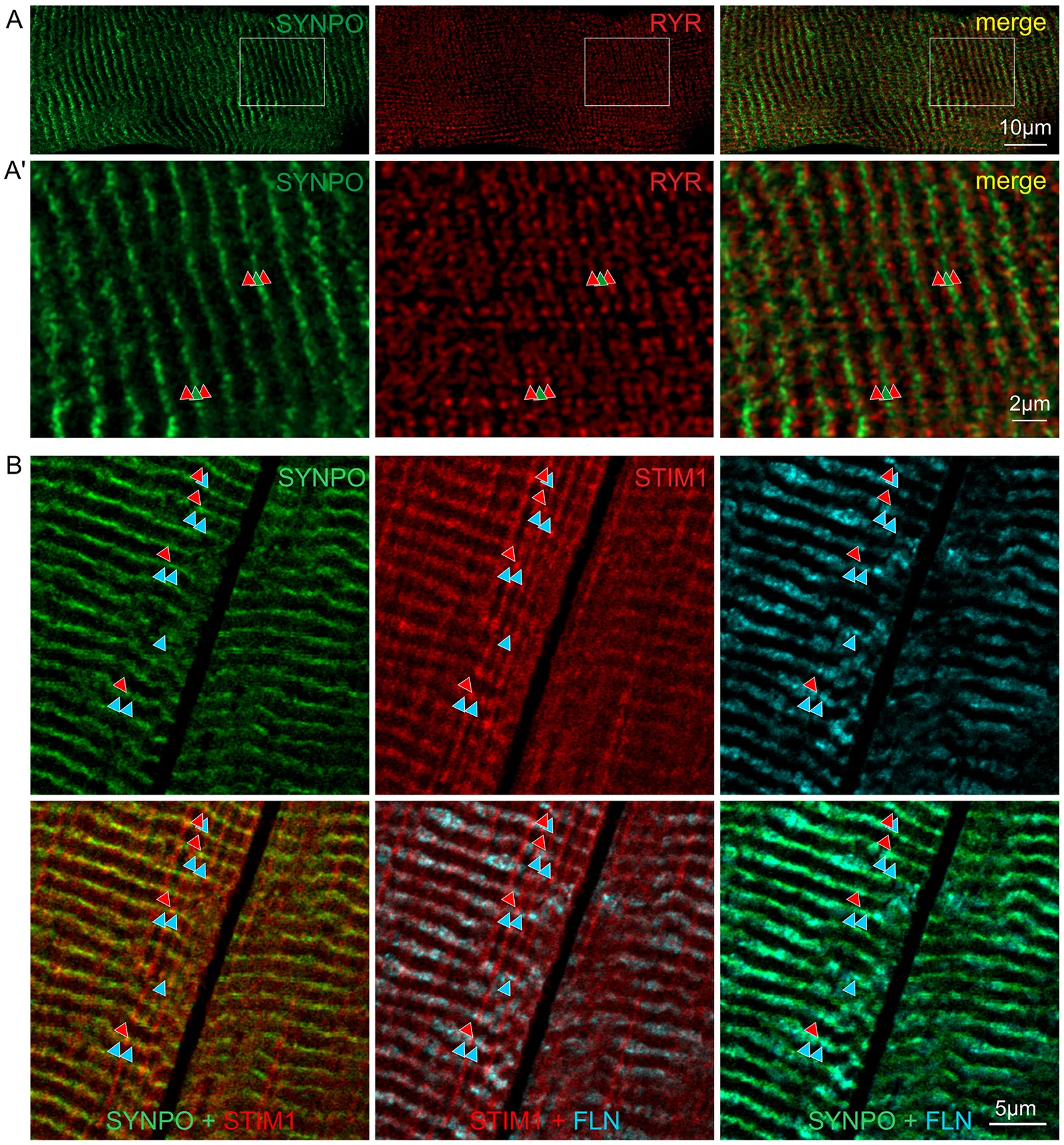
Immunolocalization of SYNPO, RYR, STIM1 and FLNc in human skeletal muscle fibers. (A) Co-staining for SYNPO and RYR results in a cross striated pattern for both proteins. While SYNPO shows the Z-disc-associated localization also shown in Fig. 3, RYR localization is clearly separated from the Z-discs and flanks the SYNPO signal on both sides. (A) Magnification of the boxed area in (A′) confirms the separate localization of RYR (red arrowheads) and SYNPO (green arrowheads). (B) Immunolocalization of SYNPO, FLNc and STIM1 (upper panel) reveals partial colocalization of SYNPO with FLNc (blue arrowheads) and with STIM1 (red arrowheads). For clarity single stainings (upper panel) and double stainings (lower panel) are shown.

**Fig. 5.**
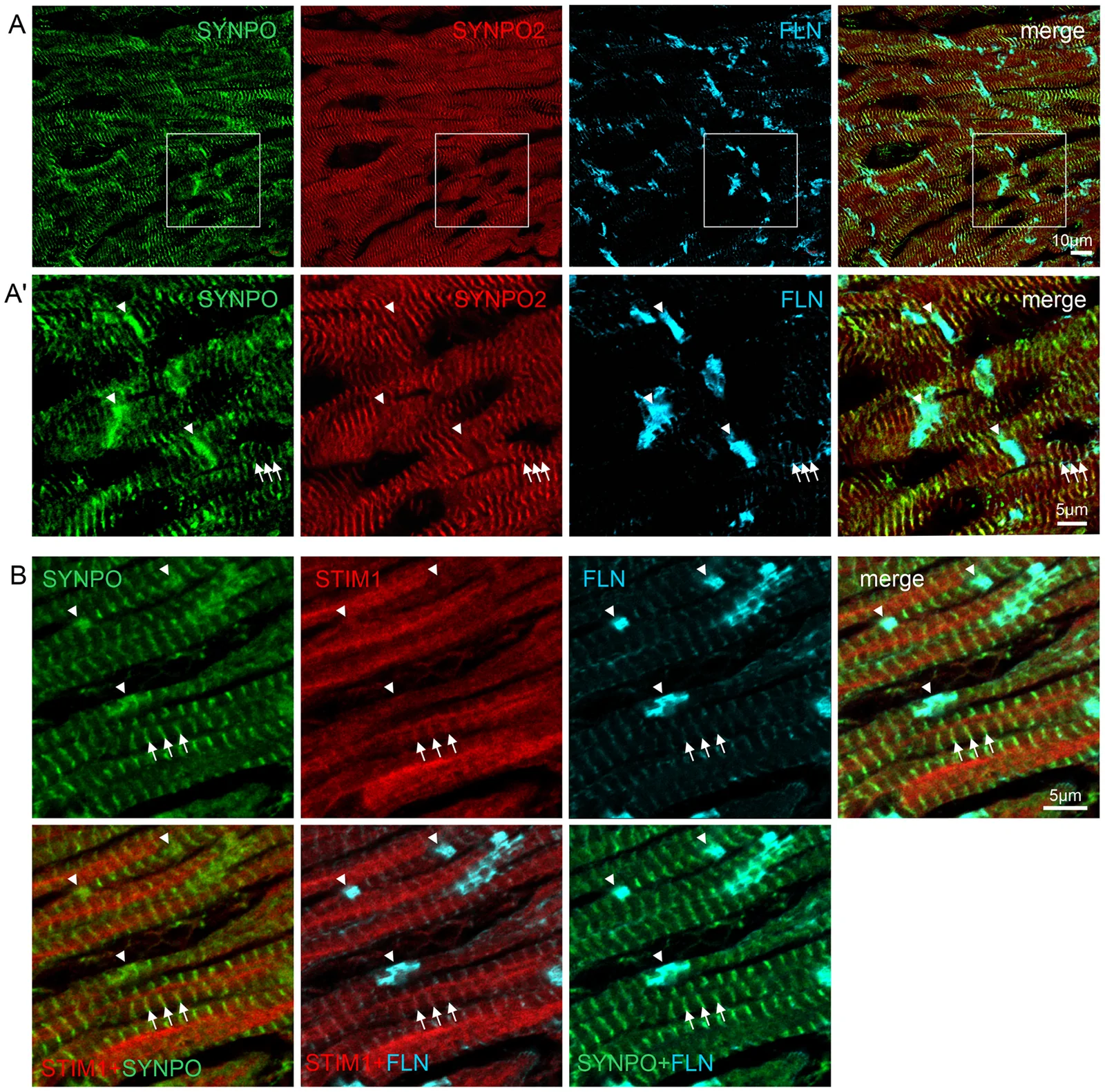
Immunofluorescence localization of SYNPO in human cardiac muscle cells. (A) Immunolocalization of SYNPO (green), Z-disc marker SYNPO2 (red) and ID and Z-disc marker FLNc (turquoise) in cryosections of human cardiac muscle. At low magnification all three proteins localize in the Z-disc area, while SYNPO and FLNc, but not SYNPO2 are found in IDs. (A′) A higher magnification of the boxed area in (A) confirms partial colocalization of all proteins in the Z-disc area (arrows), whereas only SYNPO and FLNc localize to IDs. (B) Immunolocalization of SYNPO (green), STIM1 (red) and FLNc (turquoise) in human cardiac muscle. SYNPO and FLNc both localize in Z-discs (arrows) and IDs (arrowheads). Note the more diffuse localization of SYNPO in IDs compared to the more filamentous localization of FLNc. STIM1 is only weakly associated with Z-discs and does not seem to be concentrated in IDs. Its localization is more dispersed and reticular.

**Fig. 6.**
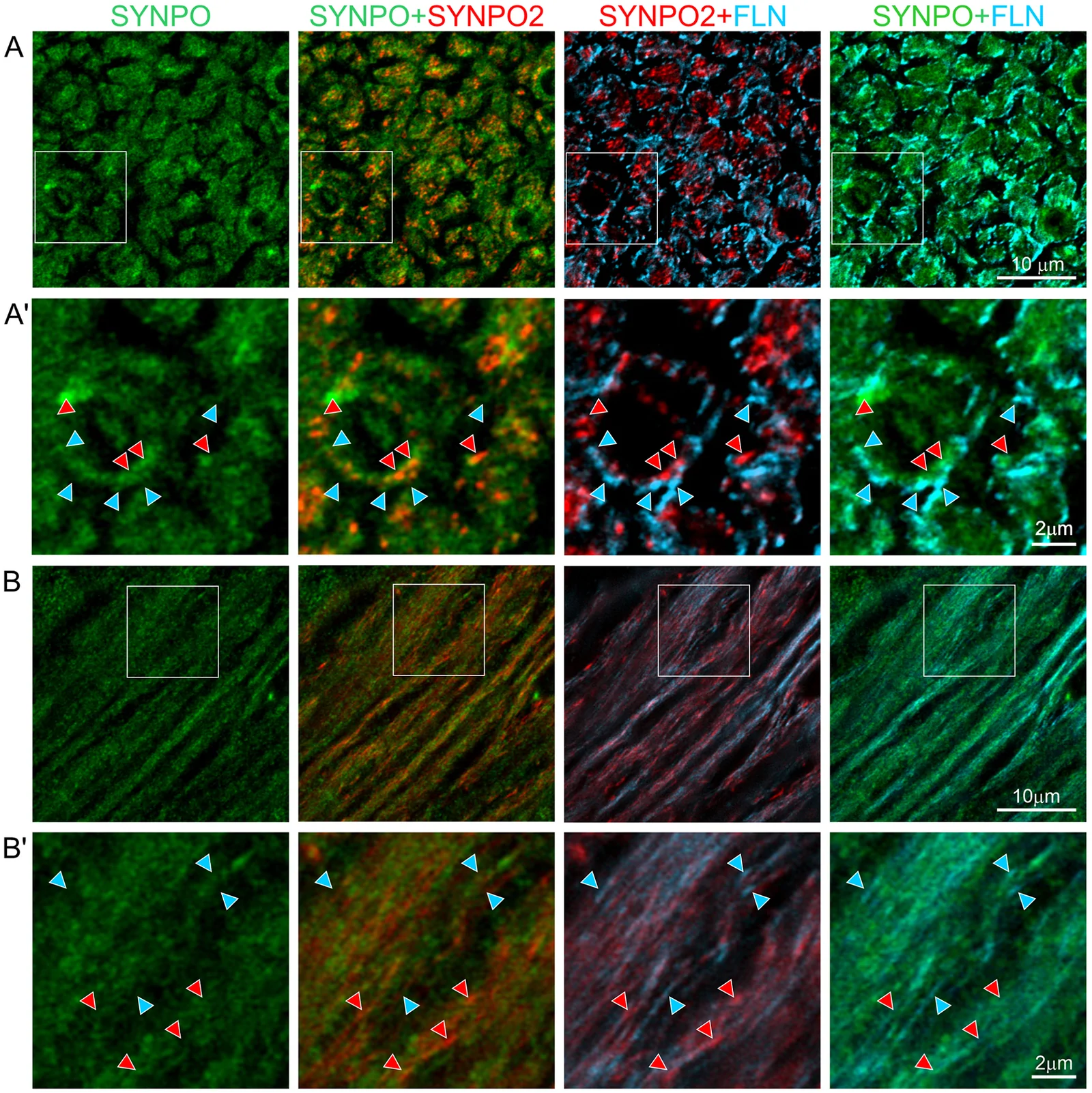
SYNPO shows a reticular distribution in human colon smooth muscle cells. (A,B) Immunofluorescence localization of SYNPO (green), SYNPO2 (red) and filamin (blue) in cryosections of human colon. A) in cross sections of smooth muscle cells of the muscularis externa of human colon, SYNPO is localized in a reticular pattern. While SYNPO is partially colocalized with filamin (blue arrowheads) and SYNPO2 (red arrowheads), colocalization of filamin and SYNPO2 is limited. In longitudinally sectioned smooth muscle cells the reticular distribution of SYNPO is confirmed. Partial colocalization of SYNPO with filamin-containing filaments or SYNPO2-containing dense bodies is observed. Boxed areas in A and B are shown enlarged in A′ and B′, respectively.

**Fig. 7.**
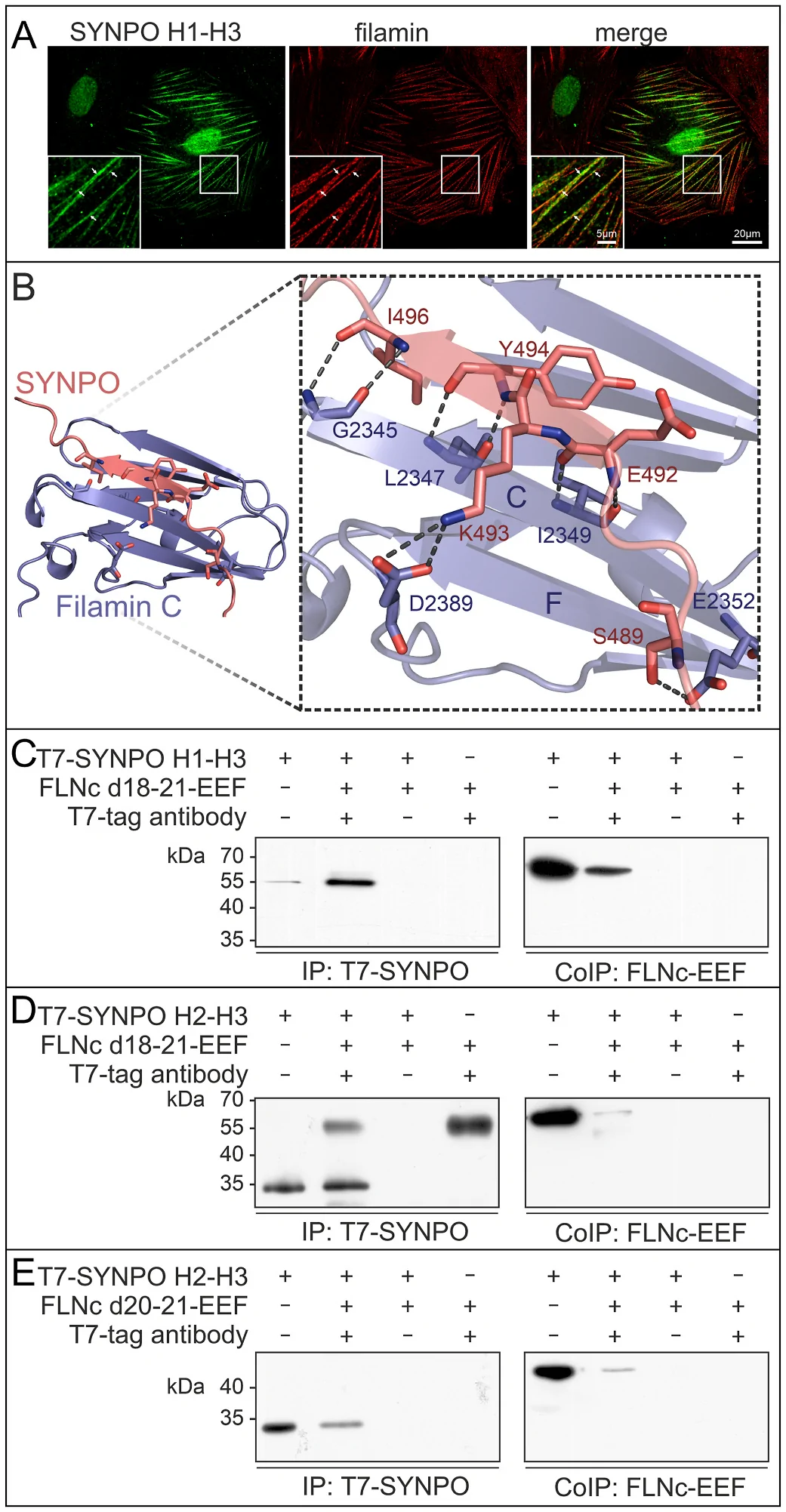
SYNPO directly interacts with FLNc. (A) Overexpression of T7-tagged SYNPO H1-H3 in A7r5 smooth muscle cells reveals targeting of SYNPO H1-H3 (green) to stress fiber dense bodies (arrows), identified by staining for filamin (red). (B) Modeling of the SYNPO-FLNc interaction by AlphaFold3 and further analysis using PyMol predicts the interaction of a small region of H2 with Ig-like domain 21 of FLNc. (C-E) Co-immunoprecipitation experiments reveal direct binding of H1-H3, the part of SYNPO, that contains the main areas showing homology to SYNPO2 and SYNPO2L, to FLNc (C) and SYNPO H2-H3 (D) bind FLNc, indicating that H2-H3 is sufficient for the interaction, while FLNc d20-21 is sufficient for binding SYNPO H2-H3 (E).

**Fig. 8.**
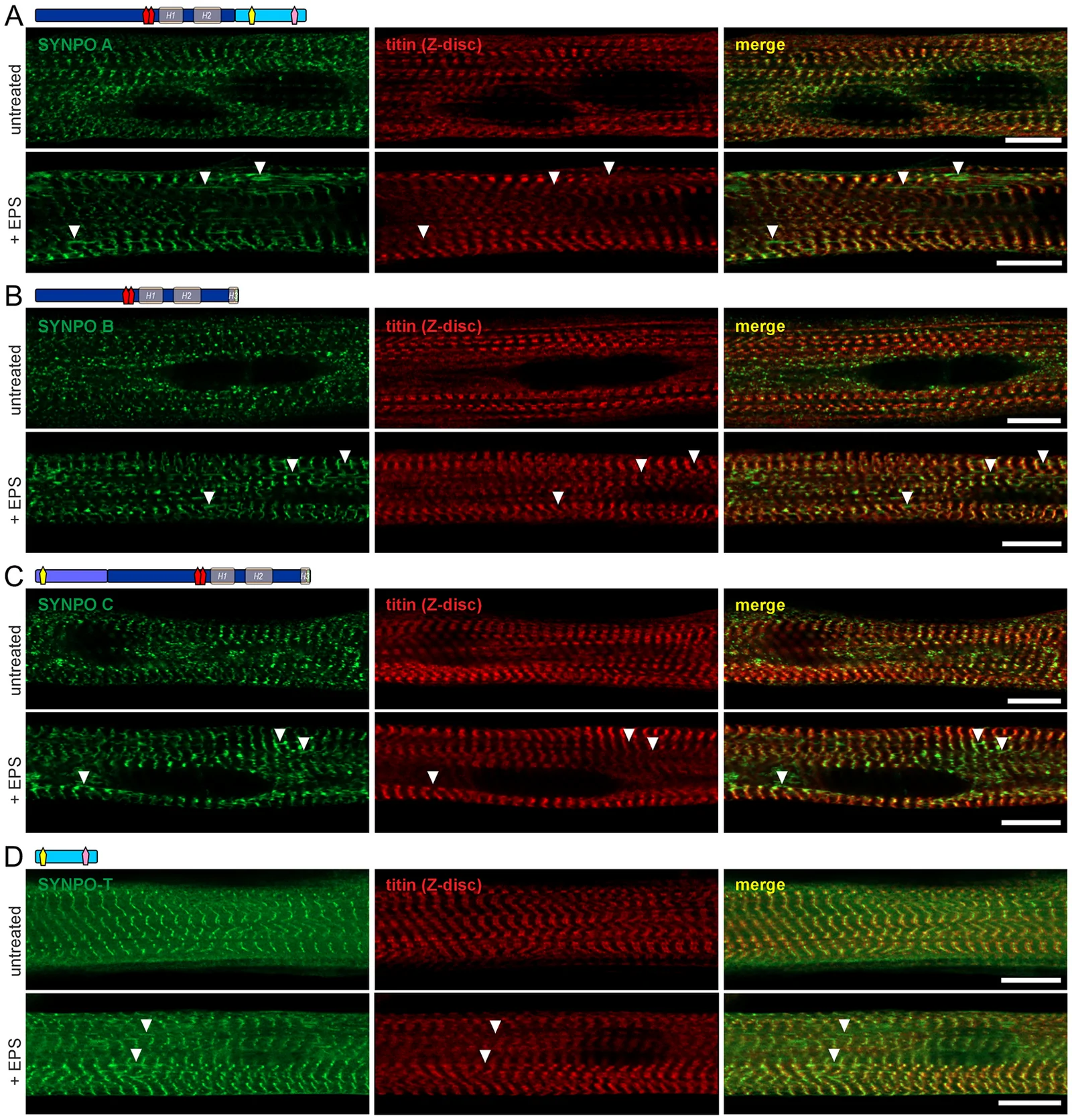
Distribution of eGFP fusion proteins of SYNPO variants upon transient expression in C2C12 myotubes with our without EPS treatment. All SYNPO isoforms localize to myofibrillar Z-discs upon transient expression in C2C12 myotubes (untreated). SYNPO-T showed in addition a more diffuse distribution, while isoforms A, B and C were also detected in vesicular structures. EPS treatment of the transfected myotubes induced a somewhat more pronounced Z-disc localization of isoforms A, B and C, while all isoforms are localized in small longitudinal structures, probably EPS-induced lesions (arrowheads). Bars: 10 μm.

**Fig. 9.**
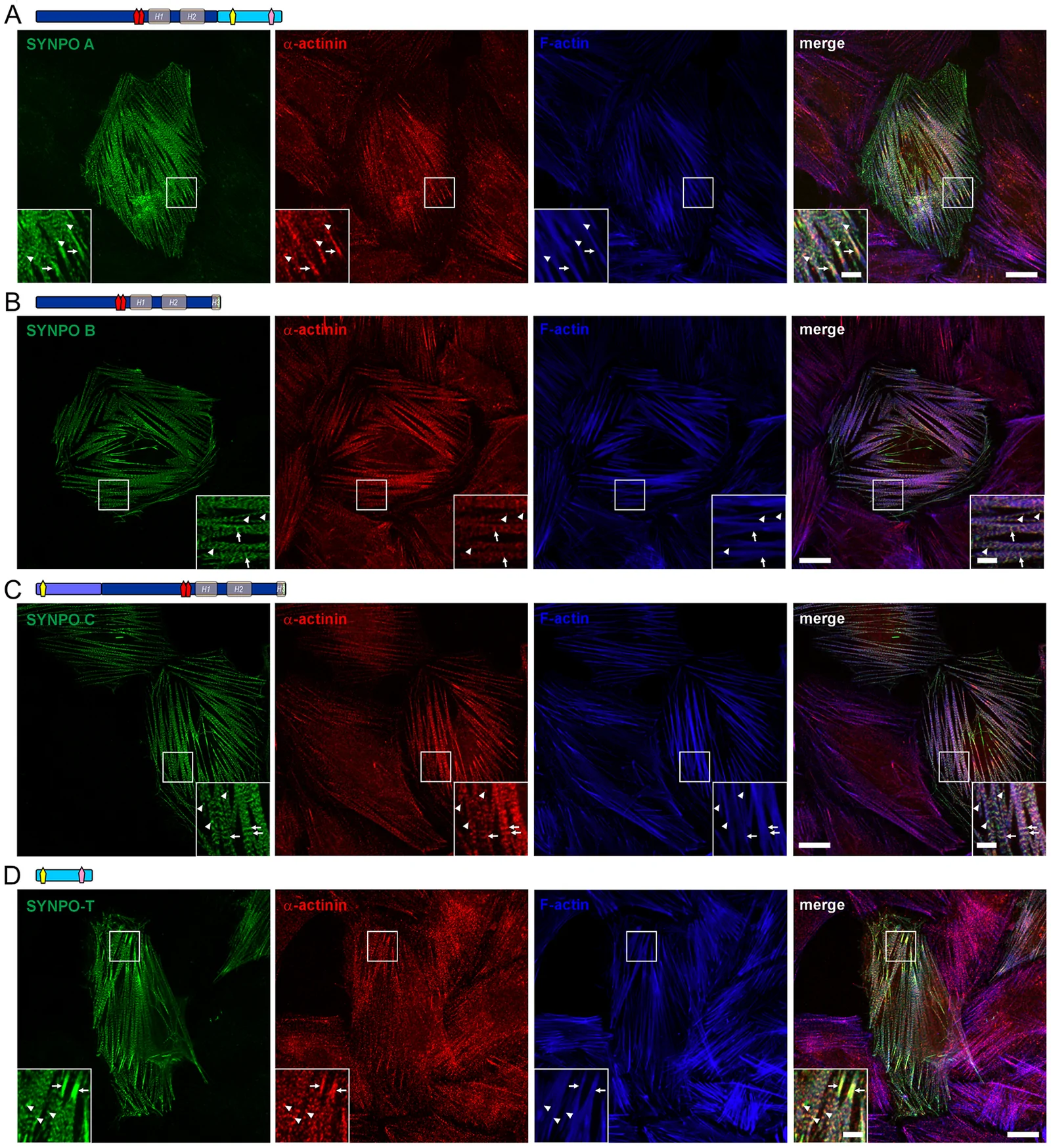
Distribution of eGFP fusion proteins of SYNPO variants upon transient expression into A7r5 rat smooth muscle cells. All SYNPO isoforms localize to dense bodies of the stress fibers (arrowheads in the insets) identified by staining with an antibody specific for α-actinin or with phalloidin, respectively. Isoform A and SYNPO-T were in addition detected in adhesion plaques (arrows). Bars: 20 μm; 5 μm (insets).

**Table 1:** Oligonucleotides used in this study. Numbers refer to the RT-PCR experiments indicated in Fig. 1 hu: human; mo: mouse.

RT-PCR	forward primer	reverse primer	Protein isoform(s)
hu 2f-4ar	CAGATGATGACAGCCAGCCC	GCTCTCCAAGGTGAACTCGT	SYNPOc, d (X1)
hu 3f-4ar	GCTGCCGATGTGGGATTTTC	TGAACTCGTTCACCCTCTGC	SYNPOa, b
hu 4af-5r	CCTTACGGCGGTGACATCTC	TCCACGAGGGAGACATGGG	SYNPOa, d (X1)
hu 4af- 4br	CCTTACGGCGGTGACATCTC	CCTTGAACCATGGACTCCCC	SYNPOb, c
huGAPDH-f-r	ATGACATCAAGAAGGTGGTGAAG	TCCTTGGAGGCCATGTGGGCCAT	-
mo 1f-4ar	CCATGGGACACCACCATTAT	GGTTGAGCTTTGGTGACCTC	SYNPOc, d (X4)
mo 2f-4ar	ACGGACGTAGCCAGGTGA	GGTTGAGCTTTGGTGACCTC	SYNPOb, d (X4)
mo 3f-4ar	GAAGAGGCCGATTGACAGAG	GGTTGAGCTTTGGTGACCTC	SYNPOa
mo 4af-4br	CGTGAGTCCCACTTACAGCA	GTGGCGTCTAAGCTCCTTTG	SYNPOc, d (X4)
mo 4af-5r	CGTGAGTCCCACTTACAGCA	GATGATGTTGCGCGCCAGAA	SYNPOa, d (X4)
mo 4cf-5r	CCTTGTCAGCAAGCTCATCC	AGGGGGACATTGGTGGAG	SYNPO-T
moGapdh-f-r	AGGTCGGTGTGAACGGATTTG	TGTAGACCATGTAGTTGAGGTCA	-

**Table 2:** Primary and secondary antibodies and other reagents used for staining. GAM: goat anti-mouse; GAR: goat anti-rabbit; GARat: goat anti-rat; AF: Alexa Fluor; HRP: Horseradish peroxidase.

antibody/ reagent	target	species	source and/or reference	dilution
Synaptopodin antibody	SYNPO (all isoforms except SYNPO-T)	rabbit	Synaptic systems 163,002; RRID: AB_887825	IF: 1:300 WB: 1:1000
G1D4	SYNPO (all isoforms except SYNPO-T)	mouse	Progen 65194; RRID: AB_2909601	IF1: WB 1:5
HH9	SYNPO2 (all isoforms)	mouse IgG1	[3]	IF 1:50; WB 1:100
RbM2	SYNPO2 (all isoforms)	rabbit	[3]	IF 1:1000
T12	titin, epitope close to Z-disc	mouse IgG1	[31]	IF 1:20
B-12	α-actinin	mouse IgG2a	Santa Cruz sc-166524; RRID: AB_2257995	IF 1:100
RR90	filamin A&C	mouse IgA	[32,33]	IF 1:50
CDN3H4	STIM1	mouse IgG1	Thermo Fisher Scientific MA1-19451; RRID: AB_2197884	IF 1:100
34C	ryanodine receptor RYR1/RYR2	mouse IgG1	DSHB; RRID: AB_528457	IF 1:5
YL1/2	EEF-tag	rat IgG2a	Invitrogen MA1-80017; RRID: AB_2210201 [34]	WB 1:700
T7-tag	T7-tag	mouse IgG2b	Novagen/Merck 69522 RRID: AB_10807769	WB 1:10,000
DE-R-11	desmin	mouse IgG1	[35]	IF 1:5
GAM IgA AF488	mouse IgA	goat	SouthernBiotech 1040-30; RRID: AB_2794376	IF 1:300
GAM IgG2a AF594	mouse IgG2a	goat	Invitrogen A-21135; RRID: AB_2535774	IF 1:500
GAM IgG1 AF594	mouse IgG1	goat	Jackson ImmunoResearch 115-585-205; RRID: AB_2338885	IF 1:300
GAM IgM AF546	mouse IgM	goat	Invitrogen A-21045; RRID: AB_2535714	IF 1:100
GAM IgM Cy5	mouse IgM	goat	Jackson ImmunoResearch 115-175-075; RRID: AB_2338712	IF 1:100
GAR Cy3	rabbit Ig	goat	Jackson ImmunoResearch 111-165-045; RRID:AB_2338003	IF 1:800
GAR AF647	rabbit Ig	goat	Invitrogen A-21245; RRID:AB_2535813	IF 1:300
GAR-HRP	rabbit IgG	goat	Jackson ImmunoResearch 111-035-144; RRID: AB_2307391	WB 1:10,000
GAM-HRP	mouse IgG + IgM	goat	Jackson ImmunoResearch 115-035-068; RRID: AB_2338505	WB 1:10,000
GARat-HRP	rat IgG + IgM	goat	Jackson ImmunoResearch 112-035-068; RRID: AB_2338134	WB 1:10,000
GAR IRdye800CW	rabbit	goat	LI-COR 926-32211; RRID:AB_621843	WB 1:10,000
PromoFluor 405 phalloidin	actin filaments	-	PromoKine PK-PF405-7-01	1:100
DAPI	nuclei	-	Sigma	1:40,000

## Data Availability

Data will be made available on request.

## Appendix A. Supplementary data

Supplementary data to this article can be found online at https://doi.org/10.1016/j.yexcr.2026.114990.
