# Supplementary material for: Characterization of synaptopodin in striated and smooth muscles: isoform spectrum, expression patterns, localization and protein interactions
Source: Exp Cell Res. Author manuscript; Available in PMC 2026 Nov 13. (PMC13759171; doi:10.1016/j.yexcr.2026.114990)
Supplement: Supplementary file 1 [file NIHMS2221975-supplement-1.pdf]

Supplementary data accompanying

Characterization of synaptopodin in striated and smooth muscles: isoform spectrum, expression patterns, localization and protein interactions

Keerthika Lohanadan[a], Marvin Assent[a], Anja Linnemann[a,1], Chengqing Qu[b], Anne Schänzer[c], Lukas Heukamp[d,2], Hendrik Milting[e], Gregor Kirfel[a], Jeffrey H. Miner[f], Dieter O. Fürst[a] and Peter F.M. van der Ven[a]

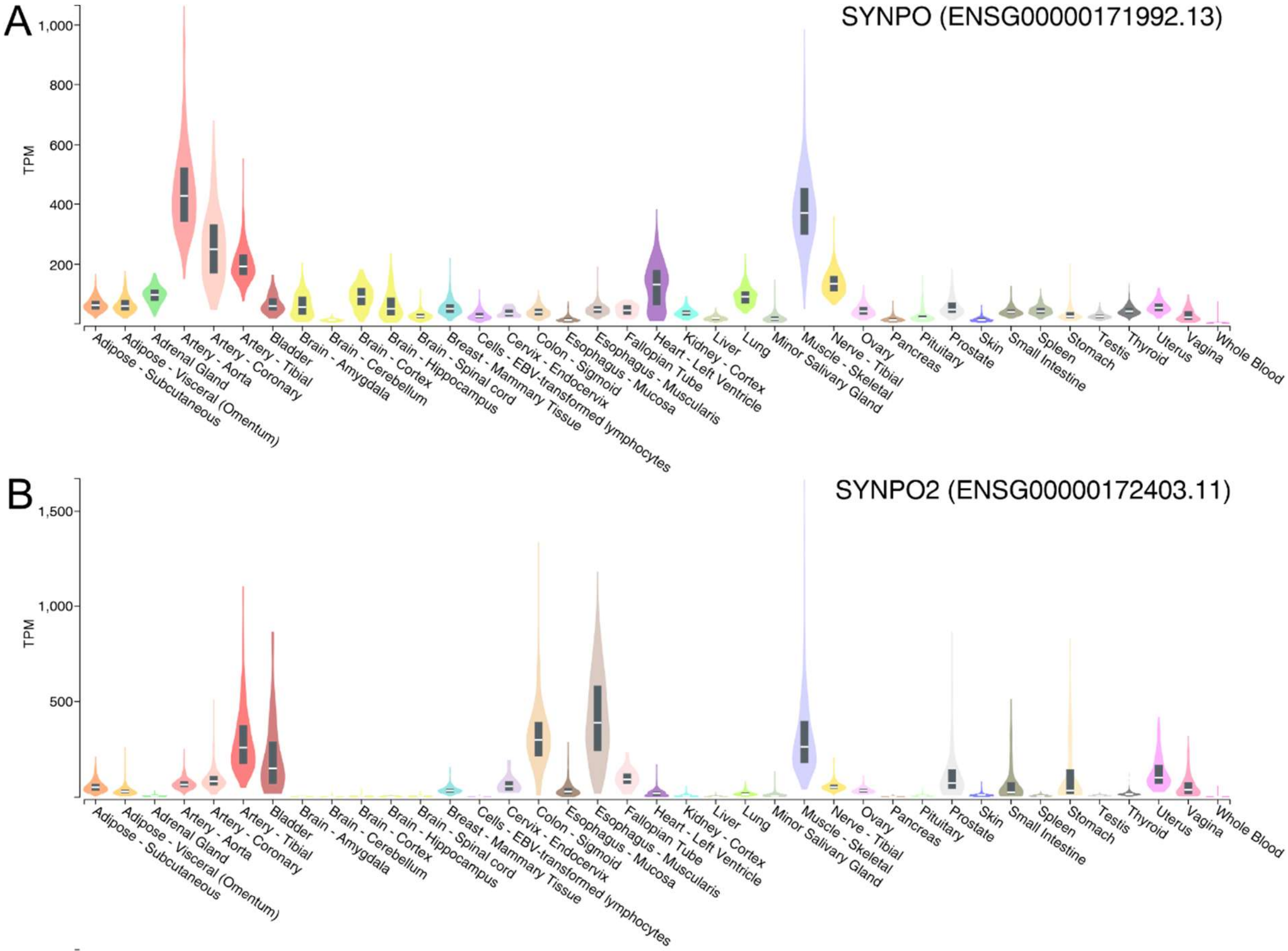

C

	heart		skeletal muscle		smooth muscle (urinary bladder)	
	HPA	GTEx	HPA	GTEx	HPA	GTEx
SYNPO	336.2	357.7	1250.7	974	28.3	54.9
SYNPO2	63.9	64.6	834.6	965.5	118	218.9

Fig. S1. SYNPO is ubiquitously expressed with highest expression in muscle tissues. Data obtained from the GTEx platform at https://www.gtexportal.org/home/ indicate that *SYNPO* is expressed in many different tissues (A). The highest expression is found in arteries, skeletal muscle and the heart. In comparison, *SYNPO2* expression is highest in esophagus smooth muscle cells, specific arteries, colon and skeletal muscle, while expression in the heart is limited (B). A quantification of the expression level of *SYNPO* and *SYNPO2* RNA in TPM in striated and smooth muscle tissue according to Human Protein Atlas (HPA) and GTEx, as indicated, confirm the data shown in panels A and B (C). TPM: transcripts per million

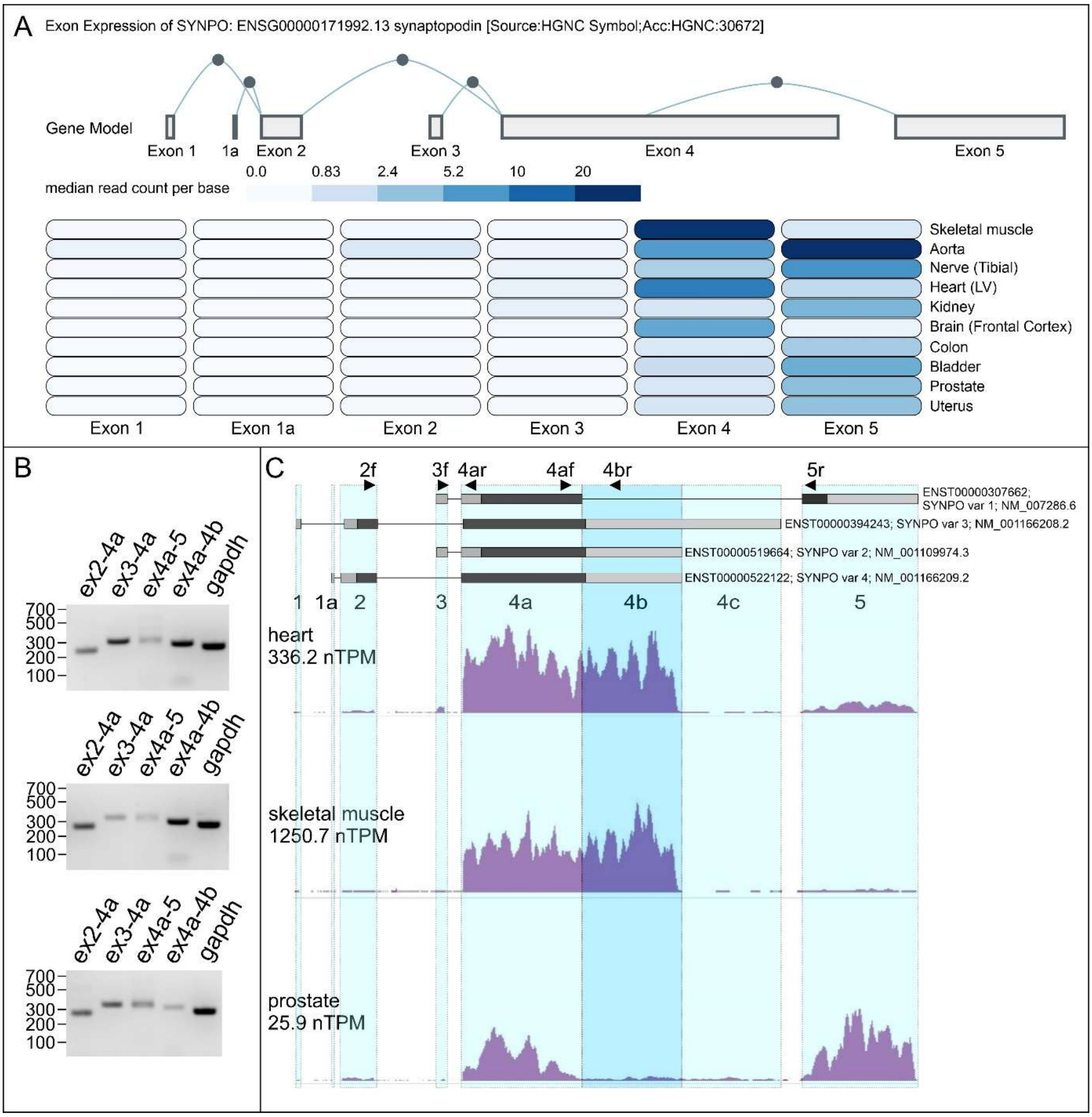

Fig. S2. Comparison of our RT-PCR data on human tissues with data from the Human Protein Atlas (v15) and GTEx on *SYNPO* exon expression.

(A) The upper panel gives the model of the human *SYNPO* gene, the potential splicing occurrences, and the expression level of the individual exons in selected tissues as analyzed by the GTEx platform. All leading exons (1, 1a, 2 and 3) are expressed at very low levels in all tissues, with highest expression in the aorta. In skeletal muscle, heart and brain exon 4 is expressed at higher levels when compared to exon 5. In all other tissues, exon 5 seems to be expressed stronger than exon 4, possibly because exon 4 is only partly contained in isoforms with exon 5. (B) The left panel shows our RT-PCR data as shown in Fig. 1B. (C) Depicted are exon-specific expression data downloaded from HPA (version 15). Unfortunately, these data are no longer available. Note that the graphs are not to scale. In all tissues, exons 1 and 2 show only weak expression. Exon 5 is mainly expressed in the prostate, with low expression in the heart.

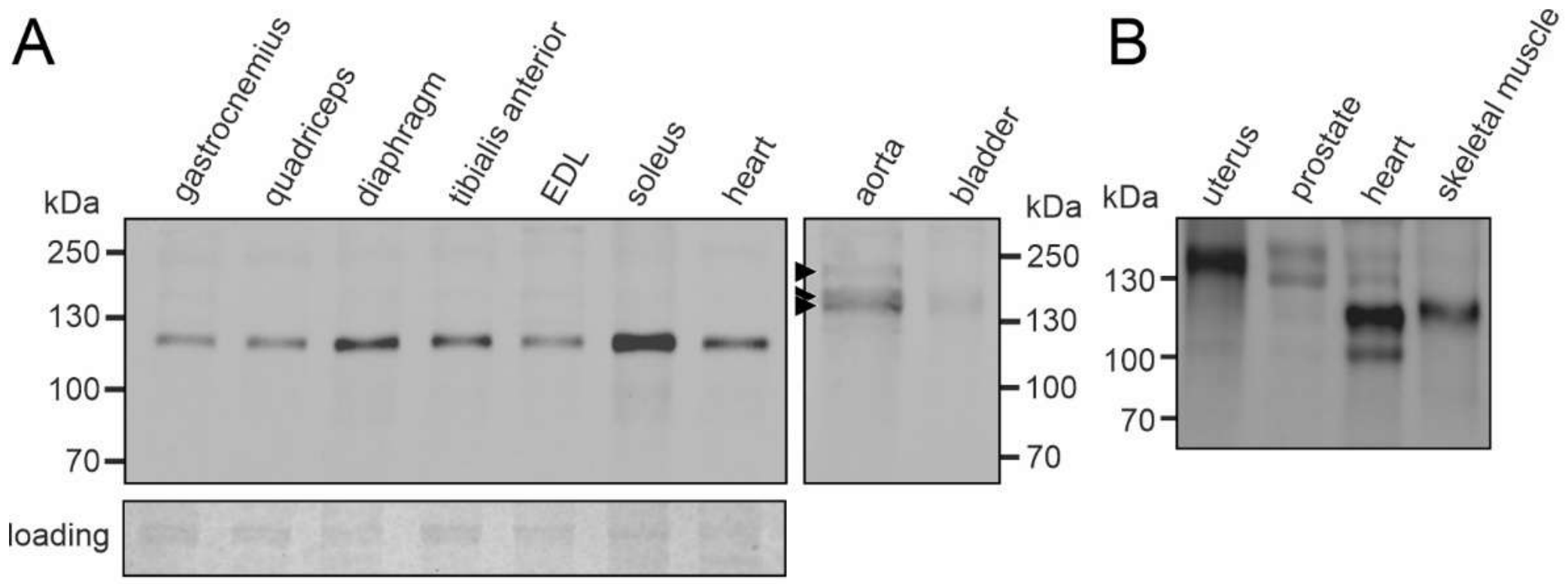

C

	HPA	GTEx
bladder	28.3	54.9
skeletal muscle	1250.7	974.0
heart	336.2	357.7
prostate	25.9	44.5
smooth muscle	37.5	–
colon	25.0	36.1
kidney	23.1	58.9
aorta	–	424.8
uterus	–	52.5

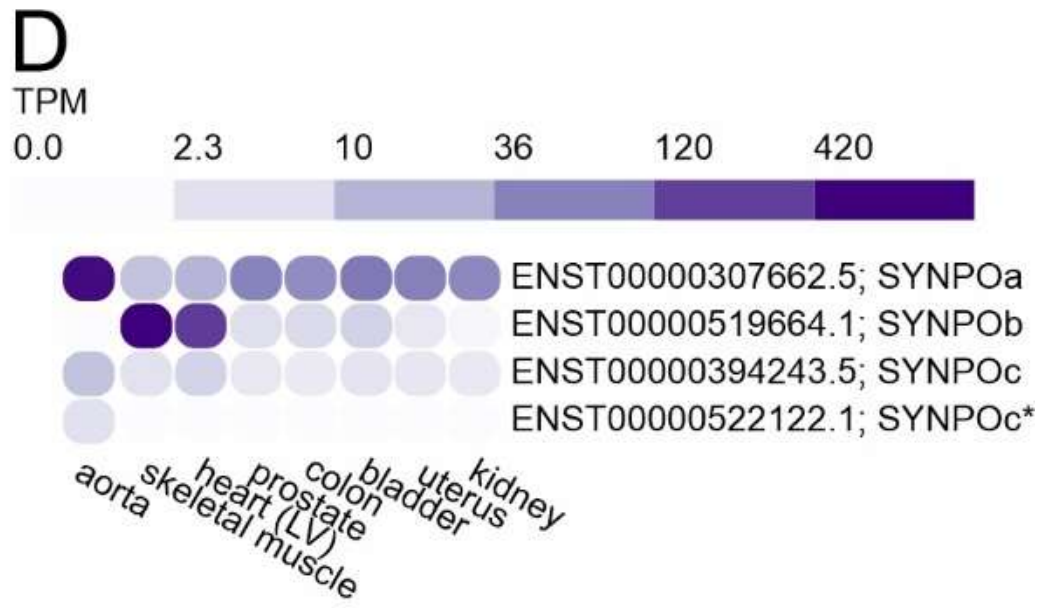

Fig. S3. Comparison of our western blot data on different tissues with mRNA expression data from the Human Protein Atlas and the GTEx portal

In panels A and B the blots from Fig. 2, showing SYNPO protein expression in different mouse (A and B, uterus) and human (B, prostate, heart, skeletal muscle) tissues are copied. For comparison, in panel C the expression of *SYNPO* mRNA (TPM, transcripts per million) in different tissues as established by HPA and GTEx is given. D gives the expression of *SYNPO* isoforms in several human tissues as established by GTEx. *isoforms ENST00000394243.5 and ENST00000522122.1 have alternative leading exons, but encode the same protein (see Fig. 1 and 2). Note that mRNA variants encoding SYNPOd are not considered by GTEx. This data confirms the main expression of SYNPOb in striated muscles, whereas other tissues analyzed here hardly express this isoform. Instead, they mainly express SYNPOa. In mouse aorta we detected three isoforms, probably SYNPOa, SYNPOc and SYNPOd.

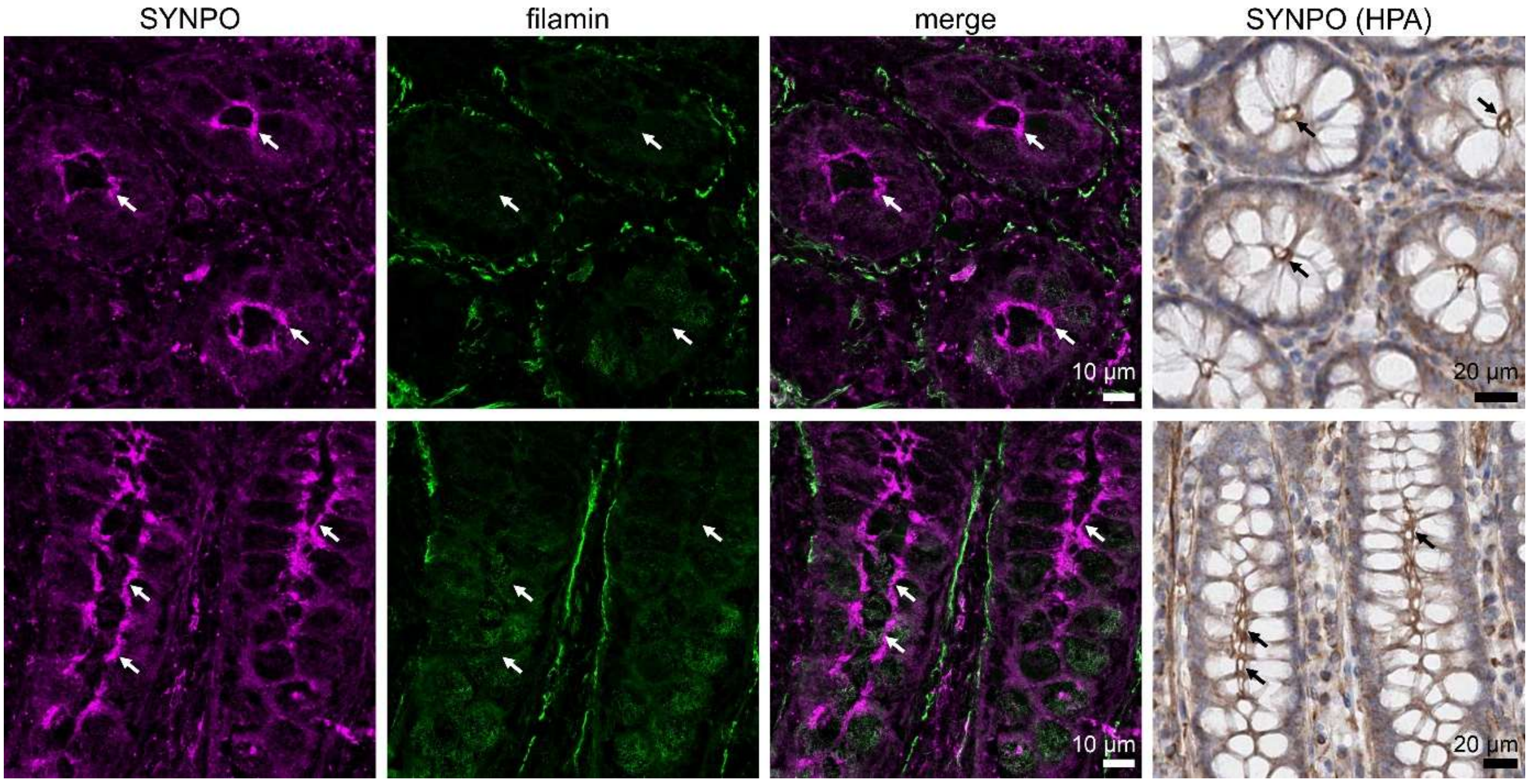

Fig. S4. SYNPO is expressed in the epithelial cells of the colon intestinal glands. Immunolocalization of SYNPO in cryosections of the human colon revealed expression of SYNPO at the apical site of the epithelial cells of the intestinal glands in both cross sections (upper panel) as well as longitudinal sections (lower panel). This staining pattern is confirmed by data from the HPA (right panels).

Table S1. Expression of SYNPO and SYNPO2 mRNA in smooth muscle cells from different human organs.

Given are the normalized expression levels of the SYNPO and (for comparison) SYNPO2 genes in smooth muscle cells according to analyses performed by the HPA (29.08.2024). nTPM = normalized transcripts per million.

Smooth muscle cells	SYNPO	SYNPO2
All tissues	**40.6 nTPM**	**287.5 nTPM**
Vascular	181.5 nTPM	1109.7 nTPM
Prostate	65.7 nTPM	288.3 nTPM
Liver	63.3 nTPM	11.9 nTPM
Breast	43.6 nTPM	226.6 nTPM
Thymus	43.5 nTPM	459.3 nTPM
Skin	40.7 nTPM	353.6 nTPM
Lung	38.8 nTPM	110.5 nTPM
Skeletal muscle	28.9 nTPM	149.3 nTPM
Esophagus	24.0 nTPM	374.8 nTPM
Salivary gland	22.0 nTPM	240.7 nTPM
Tongue	21.0 nTPM	135.0 nTPM
Fallopian tube	20.4 nTPM	457.6 nTPM
Ovary	16.3 nTPM	117.3 nTPM
Placenta	14.9 nTPM	57.3 nTPM
Adipose tissue	14.9 nTPM	223.4 nTPM
Endometrium	10.5 TPM	284.9 nTPM
